# Inclusion of Refugees Into National Health Systems: Enablers, Barriers and Lessons From Six Country Case Studies

**DOI:** 10.1002/hpm.70109

**Published:** 2026-07-23

**Authors:** Sophie Witter, Alastair Ager, Karin Diaconu, Zahid Ali Memon, Ben Ngoye, Natasha Palmer, Marcia Vera‐Espinoza, Amina Olabi, Hussam Rekani, Hassan Ould Moctar, Ricardo Chuquimia, Chitalu Miriam Chama‐Chiliba, Ibrahim Bou‐Orm, Maria Paola Bertone

**Affiliations:** ^1^ Institute for Global Health and Development Queen Margaret University Edinburgh UK; ^2^ Department of Community Health Sciences Aga Khan University Karachi Pakistan; ^3^ Strathmore University Nairobi Kenya; ^4^ Independent researcher Duhok Iraq; ^5^ School of Oriental and African Studies (SOAS) London UK; ^6^ Independent researcher Lima Peru; ^7^ Department of Economics University of Zambia Lusaka Zambia; ^8^ Liverpool School of Tropical Medicine Liverpool UK

**Keywords:** governance, health financing, health information, health system integration, LMICs, refugee inclusion, service delivery

## Abstract

The global refugee population has nearly doubled in the last decade, and many of these populations face chronic displacement, often in neighbouring countries with limited resources. Emergency, short‐term responses are being transitioned to longer term approaches, including a focus on inclusion through coverage in national health systems. However, analysis of empirical and comparative evidence on how to enact these approaches has been limited. In this article, we analyse the inclusion of refugees in six low and middle‐income country (LMIC) health systems (Kenya, Kurdistan Region of Iraq (KRI), Mauritania, Pakistan, Peru and Zambia). Using a shared framework and tools, we collected data over 2023 to 2025, using document reviews, key informant interviews, focus group discussions and participatory workshops. Analysis was carried out separately for each setting, validated locally, and later compared for synthesis. We focus on key health system domains—governance, health financing, service delivery (including the health workforce) and health information systems—outlining the attributes of refugee inclusion and system integration in each, identifying patterns across the case studies and barriers and enablers. We identify leverage points for improving integration but highlight that inclusion of refugees into national health systems is complex and does not guarantee improved outcomes, especially when refugees previously benefited from a higher level of resources and are being included in strained host health systems. However, committed government leadership, coordinated international support, and multi‐year investments in strengthening key components of the local health system, including enabling refugee health staff to engage effectively in it, can make the transition to an integrated system a beneficial one for both refugee and host communities.

## Highlights

- This paper adds much‐needed empirical analysis of health system integration processes for refugee populations across six LMIC settings
- Integration has many attributes, which are interconnected but not all equally urgent. In health financing, for example, distinct revenue streams may not be problematic if risk pooling is effective
- Good outcomes from health system integration are not guaranteed: they depend on investments in strengthening local health systems, which in turn are enabled by policies of domestic resource mobilisation, improved aid coordination, refugee health worker inclusion, effective decentralisation and strengthened government leadership, refugee inclusion in sectoral governance structures and harmonised data gathering and use
- Performance on refugee inclusion and system integration varied across the case studies, often driven by factors external to the sector, such as economic conditions and political framing. Shocks can also play a role, and recent aid cuts are one such shock, shifting the landscape globally

## 1 Introduction

In mid‐2025, 117.3 million people were recorded as forcibly displaced globally, with 36.4 million of these being refugees. The numbers of displaced people have been growing every year since 2011, nearly doubling in the past decade, and the bulk of these (73%) have been displaced to low‐ and middle‐income countries (LMICs), which by definition have limited resources to accommodate them. Conflict and war are key drivers of displacement, often over prolonged periods. Meanwhile, the international system which was developed since the Second World War to minimise conflicts and support refugees and other forcibly displaced populations is under unprecedented pressure, with cuts to aid funding which threaten health and development gains [1] and geopolitical tensions which threaten global cooperation.

This challenging background increases the urgency of understanding how best to accommodate refugees in their host countries, particularly after the immediate emergency period of displacement, in a way which is effective and sustainable. The Global Compact on Refugees, endorsed by 181 countries, supports inclusion of refugees into national health systems and calls for enhancement of health service quality and coverage to benefit refugees and host communities [2]. UNHCR's global public health strategy calls for inclusion of refugees and stateless people in national health policies, plans, programmes, national statistics and budgets, enabling equitable access to health services on a par with nationals [3]. While this shift away from parallel provision has normative traction, studies which examine how to proceed with inclusion within local health systems are relatively few and have highlighted the need for more empirical research on refugee inclusion and health system integration processes and outcomes [4, 5, 6].

Based on six case studies carried out in selected LMIC settings (Kenya, Kurdistan Region of Iraq (KRI), Mauritania, Pakistan, Peru and Zambia) over 2023 to 2025, this article presents lessons on managing refugee healthcare inclusion in local health systems. Our cross‐country case study analysis identified common enablers, barriers and leverage points that can sustain refugee inclusion and health system integration processes in different LMIC contexts.

## 2 Methods

This paper is part of a research programme funded by UNHCR. An initial global literature review was conducted in 2023 on refugee inclusion and integration of national health systems [5, 7] to assess the state of evidence and its gaps. The research team then developed a conceptual framework (Figure 1) to structure six country case studies, along with a common research protocol, which was adapted in each case.

**FIGURE 1:**
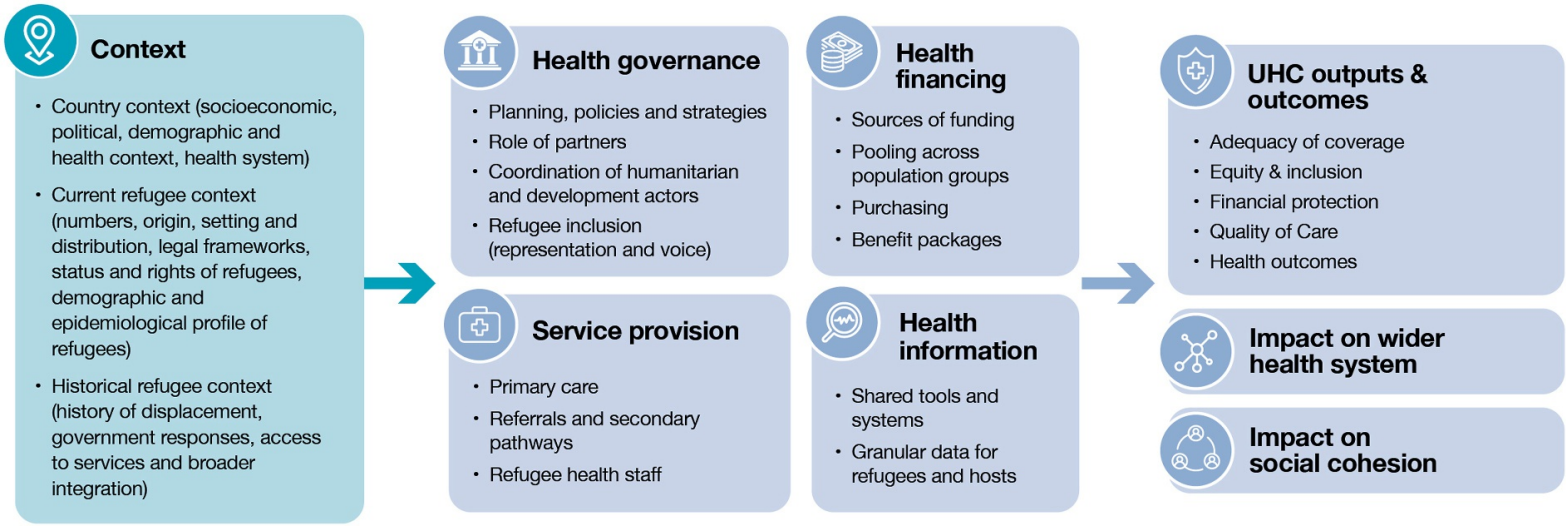
Conceptual framework for research. *Source:* Authors.

The country case studies adopted a mixed‐methods, exploratory and participatory, retrospective design and were undertaken over 2023–2025. Country case studies were analysed and validated with country partners. In a second phase, comparative analysis was undertaken by the research team. The first output of this comparative analysis was a paper analysing the policy trajectory across the six countries, which focuses on how the context factors (in the left side of our conceptual framework) influence decisions relating to and sequencing of health system integration [8]. That paper lays out the refugee characteristics, their phases of displacement and origins, as well as legal status and country contexts, through a short summary is also provided in this paper in Supporting Information S1. This paper focuses on the central section of our conceptual framework—the health system components, their integration (or not) and how these influence the outputs, outcomes, systems and community effects on the right‐hand side of the framework. Discussion of the two‐way relationship between system integration for refugee inclusion and social cohesion will be the subject of a third comparative paper [9].

Ethics approval for primary data collection for all case studies was obtained from Queen Margaret University (UK). Additional ethics approval was obtained from Strathmore University (SU‐ISERC2381/24), from the National Health Research Ethics Board in Zambia (NHREB003/10/08/2023) and from the Aga Khan University in Pakistan (2023–9185‐26641). Additionally, clearances were obtained from health and administrative authorities in the KRI, Zambia and Pakistan. No additional country ethics approval was required for Peru and Mauritania.

### 2.1 Country and Site Selection

The six case studies focus on Kenya, Kurdistan Region of Iraq (KRI), Mauritania, Pakistan, Peru and Zambia. The selection was guided by UNHCR's specific policy and operational interests (e.g., in Kenya and Zambia, there was an interest in understanding how engagement with the national health insurance processes might affect refugee inclusion), with an overall focus on LMICs, ensuring representativeness of geographic regions, and avoiding duplication with settings that are well covered in the literature. In each country, the population of focus was selected to represent the people covered by UNHCR's mandate. Throughout the paper, we use the term ‘refugees’ in reference to the people covered by UNHCR's mandate, which includes not only refugees whose status has been recognised but also asylum seekers and those with other statuses determined by complementary pathways or local legislation (e.g., to include Venezuelan migrants in Peru).

Two study sites were chosen in each country in order to capture appropriate diversity of circumstances. This was done in consultation with the UNHCR country office and typically consisted of an urban site (often the country capital), and a second site, in a more rural area or smaller town, where refugees are often (though not in all cases) settled in a camp or village (Table 1). In KRI, a third site was selected to ensure all governorates were represented (excluding Halabja/Helebçe where the number of refugees is low), each with a mix of urban and camp settlements. For details of the sites and contextual information on the countries, their refugee settlement patterns, health systems and policy trajectory on refugee integration, see [8].

**TABLE 1:** Summary of data sources for country case studies.

Description		Kenya	KRI	Mauritania	Pakistan	Peru	Zambia
Document review	Articles and grey literature	27	53	45	82	45	55
KIIs/(FGDs)	Governmental representatives	4 (+2 FGDs)	12	2	10	14	9
	Funders and development partners	5	5	7	7	7	3
	NGOs/iNGOs and implementing agencies	3	5	9	9	4	6
	Health providers	5 (+1 FGDs)	2	2 (+2 FGDs)	—	3	5
	Refugee and host community, CSOs	—	4	1 FGD	4	2	9
	TOTAL KIIs/FGDs	17 KIIs 3 FGDs	28 KIIs	20 KIIs 3 FGDs	30 KIIs	30 KIIs	32 KIIs
Group model building sessions	At central level (no. of participants)	—	—	—	1 (14)	1(10)	1(6)
	At study sites (no. of participants)	2 (21 + 6)	2 (10 + 17)	2 (12 + 11)	4 (9 + 8; 4 + 3)	2 (15 + 11)	1 (10)
	Total participants in GMBs	27	27	23	38	36	16

### 2.2 Data Sources

Fieldwork was carried out in two waves (late 2023‐early 2024 for Zambia, Mauritania and Pakistan; and mid 2024‐early 2025 for Peru, KRI and Kenya). Table 1 provides a summary of data sources and details of the methods are given in Supporting Information S2 (study protocol).

For each setting, data sources included a documentary review, secondary analysis of existing financial and health datasets, as well as a series of key informant interviews (KIIs) and/or focus group discussions (FGDs). Documents and datasets included peer reviewed articles, grey literature such as government policies, plans and guidelines, reports by external partners, online media reports, and datasets (where available) from routine information system, operational or surveillance data and surveys. They were retrieved through purposive internet searches, focussing on relevant organisational websites as well as databases (PubMed and Google Scholar), as well as shared by the UNHCR country offices and other key informants. KIIs and FGDs included participants involved at different levels in refugee health service provision, such as government representatives, funders and development partners, national and international NGOs, health providers, and refugee and host community representatives. Participants were identified by means of a purposive sampling approach, carried out through existing contacts facilitated by the local UNHCR office and local researchers, and informed by the document review. The choice between individual interviews and FGDs was made depending on the circumstances of the interview and the preference of the participants, who in some cases were more comfortable with group interviews (e.g., facility staff). Interviews and FGDs followed a standard topic guide adapted for each settings [8]. Some interviews were conducted virtually, via Microsoft Teams, especially with actors at central level, while interviews at local levels were done in person. Interviews were conducted in English, Swahili, French, Arabic, Kurdish and Spanish depending on the preference of the participants. After reviewing the participant information sheet and addressing any questions, consent was taken in writing or orally. Interviews were recorded where possible and transcribed, and detailed notes were taken. Transcripts and notes were anonymised using alphanumerical codes before analysis.

In addition, to complement the interview data collection, in each setting 2‐3 Group Model Building (GMB) workshops were convened (one for each study site), bringing together refugee and host community representatives, local government, service providers and implementing agencies. Where possible, an additional GMB workshop was convened with central level policymakers. The aim of the GMBs was to engage stakeholders in participatory discussions on strategies, barriers and entry points for successful inclusion of refugees in the national health system in each setting. Each of the workshops was structured with respect to specific group model building activities [10, 11]. These involved eliciting understandings of change over time regarding provision of healthcare to refugees in that setting, identifying variables that had influenced that provision, constructing a causal loop diagram (CLD) showing linkages between them and, finally, reflecting on the causal loop model and suggesting leverage points for potential action to strengthen integration of provision.

### 2.3 Data Analysis and Synthesis

Data was analysed thematically using the conceptual framework. Within each case study, data analysis of different sources was done iteratively, to allow for triangulation of information. Six stand‐alone reports were developed based on the case studies, which integrated data sources, which we draw on for this paper [12, 13, 14, 15, 16, 17]. These were shared with country, regional and global UNHCR partners for checking and validation. Findings from the six case studies were mapped, compared and contrasted during comparative analysis workshops held in June 2024 and April 2025.

## 3 Findings

In the findings, we examine four health system areas in turn—health governance, health financing, service delivery (including health workforce), and health information systems, which were selected for focus in this study. For each, we start by outlining what inclusion and system integration for refugees means in relation to this area (what the attributes and choices around integration are in this domain). We then highlight the patterns found in the case study countries (Supporting Information S1 provides more details), enablers and barriers to inclusion and system integration, and lessons arising, in terms of the importance of this area and its effects on our outcomes of interest. All the findings here draw from the six country reports. As highlighted above, we have not focused on findings on social cohesion as that is the subject of a separate paper [9].

### 3.1 Governance

#### 3.1.1 Attributes of Health Governance Integration and Refugee Inclusion

Integration is a term most commonly applied to service delivery, however, it is also relevant for governance, where integration can take different forms to enable refugee inclusion. All of these are normatively positive, as they involve breaking down silos and increasing inclusion, and therefore have the potential to promote process equity as well as efficiency and effectiveness in the long term. Here we consider the following three aspects:

- Whether national health policies, plans and strategies explicitly consider refugee health needs and plan for them together with the host population
- Whether refugees are included in the development of plans for services which affect them
- Whether there is effective coordination across actors supporting refugee and host population health care

#### 3.1.2 Patterns in Case Study Countries

##### 3.1.2.1 Policies and Plans

In relation to inclusion of refugees into national policies and strategies, the case study countries are at different stages, ranging from advanced integration in Zambia to full separation and parallel planning in Pakistan. Kenya has plans for full inclusion, but these have not yet reached the stage of integrated plans at national level, though at county level some areas have developed operational plans for refugee inclusion and system integration. KRI has developed inclusive policies and practices after the initial emergency phase (since 2015), but without any legal framework for refugee inclusion, which creates a risk of political attention dropping in future. In Peru, a legal framework was also lacking, and the unit formulating and promoting policy guidelines, plans and programmes for refugees and migrants within the Ministry of Health lacked a permanent staff or budget. Interviewees and GMB participants from Peru noted that many policies do not explicitly list refugees and migrants and this in turn influences the types of services which facilities make available. In Mauritania, a recent roadmap points towards refugee inclusion and system integration but this is not yet reflected in national plans. Urban refugees in some settings like Mauritania appear to be more overlooked in policies and programme, due to their dispersion in the population (rather than being living in camps).

##### 3.1.2.2 Refugee Inclusion

Refugee inclusion in decision making on policies and services at national level was weak across all of the case study countries, though the need for it was generally recognised. For example, this is considered a priority of the 2024 Refugee Policy in Zambia, where refugee participation is called for in all the fora where decisions are made regarding refugees, as also in the 2021 Refugee Act in Kenya. At local levels, refugees are included in local governance structures, such as health committees in Mauritania and community boards in KRI. In Peru, community organisations which represent refugees are active in programme implementation for health and other services, linking communities with service providers.

##### 3.1.2.3 Effective Coordination

Coordination structures were perceived as functioning relatively effectively across the case study countries, although structures varied. Zambia adopted a government‐led multisectoral approach, while in KRI, routine government‐led health sector mechanisms are used to coordinate all of the stakeholders involved in refugee health care. In Kenya, coordination is led by the Department of Refugee Services, co‐chairing with UNHCR at different levels of the system, with the MoH co‐chairing for health, while Peru has created platforms for actor coordination which sit outside government, and Pakistan's Commissionerate for Afghan Refugees coordinates in parallel to other health sector mechanisms.

#### 3.1.3 Enablers and Barriers

Regarding the integration of governance functions in relation to health, a number of important enablers emerged from the case studies. One was legal provisions which are inclusive. For example, the 1993 constitution in Peru enshrines the right to health protection (Article 7) and guarantees free access to health services (Article 11). Specifically for refugees and migrants, the Migration Law's regulations (Article 7) mandate that the Ministry of Health ensures access to public health services, even for irregular migrants. However, while this is supportive, it is not sufficient: the Ministry has yet to issue clear regulations to organise or guarantee this care, leading to inconsistencies in access. Equally, in Zambia, participants noted that the 2017 Refugee Act and the 2024 Refugee Policy provided a framework that facilitated action and further allowed diverse stakeholders to come together but also hold each other to account. International norms were also cited as supportive in the case studies, specifically the Global Compact on Refugees.

Decentralisation can also be supportive of more integrated approaches, as highlighted in the Kenya case study, where county governments now have sufficient decision space to be able to develop and deploy integrated planning, though again this is not a guaranteed outcome. Health as a sector is clearly strongly affected by wider administrative reforms, which can be supportive in enabling local governments more control over tax‐raising and social service provision, and partners like the World Bank have supported integration in that context in settings like Kenya.

Adopting a whole of society approach appears to have been beneficial in mobilising and sustaining action for integration, for example in Zambia or Peru, where UNHCR advocated frequently for all services offered to refugees to also be offered to host communities, which aided the argument that health provision in settlements should be assured by the government and also helped make the case for additional services.

Very practically, transfer of NGO facilities into public ownership, as happened in Mauritania and Kenya, where from 2020 the camp facilities have been overseen by the district health team and added to the local health plans and budgets, is a key step and enabler in more integrated governance. This needs to be accompanied by shifting roles of key international actors such as UNHCR. UNHCR's ability to adapt its engagement has been a key enabler in a number of settings. For example, in KRI, as the government took more leadership, it shifted from providing financial resources and establishing services during the early phase to providing technical support, capacity building and monitoring, along with more targeted financial support (such as filling occasional gaps in medical supplies, supporting mental health programmes, and advocating for refugee inclusion in emerging health insurance or social protection schemes). Similarly, in Zambia, UNHCR initially acted as purchaser and coordinator of services, directly ensuring health service delivery by implementing partners during the emergency phase. However, in the post‐emergency phase, UNHCR stepped back from this role and advocated for the central Zambian government to take on responsibilities for refugees.

Although not highlighted very explicitly in the case studies, barriers to integration of the governance functions include a number of contextual factors which we highlight in our policy trajectory paper [8], including perceptions of overload in relation to refugee numbers, hostile geopolitical factors and political rhetoric which provides a negative framing of refugee populations, along with host health system weaknesses, which in the case of Pakistan are amongst the factors behind the limited health governance integration.

### 3.2 Financing

#### 3.2.1 Attributes of Health Financing Integration

For health financing, integration can be considered in relation to the main functional domains, as laid out by WHO [18]. These functions are distinct but often inter‐connected, and some may be more important (or more urgent) in relation to refugee inclusion and integration of national health financing systems.

- For revenue raising, integration would imply that resources for refugees and hosts are drawn from the same sources
- For pooling, integration relates to refugees and host populations sharing risks, being included in the same protection schemes and therefore cross‐subsidising one another
- For purchasing, integration indicates shared contracts for services and possibly shared payment systems for services covering both population groups
- In relation to benefits packages, having the same entitlements and facing similar costs to accessing them across the two groups would be the main features of integration.

#### 3.2.2 Patterns in Case Study Countries

##### 3.2.2.1 Revenue Raising

Across the countries, revenues sources and the balance between them varies considerably, with most funding for refugee health care coming from domestic sources in KRI, Peru and Zambia, while all other settings draw on a mix of international and domestic sources. Patterns in part follow national income levels, with middle income countries more likely to be self‐financing, though size of refugee population is also a factor: Zambia draws largely from domestic sources despite being low‐income, while funding streams remain parallel in Pakistan, where the refugee population is much higher.

Development funding (rather than humanitarian funding) is playing an increasing role in contexts such as Kenya and Mauritania, which supports more integrated approaches, as these funds are typically earmarked for host and refugee communities. For example, the BREHS (Building Resilient and Responsive Health Systems) project in Kenya, supported by the World Bank, focuses on system strengthening for refugees and host communities in Garissa and Turkana and funding will be channelled as much as possible through public systems (public finance mechanisms, the Kenya Medical Supplies Agency (KEMSA) for procurement, and county payroll for staff, e.g.). Resource constraints and reductions in funding are noted in many of the case study countries, such as KRI, Mauritania, Zambia and Pakistan.

##### 3.2.2.2 Pooling

In Zambia, KRI, Peru, refugees and host communities have pooled risks in terms of accessing public services supported by public budgets (Zambia and KRI) or having equal access to social health insurance (SHI) schemes (Peru), with any additional support from UNHCR and other funders benefiting both communities through system strengthening. Other case study countries are moving in this direction, in planning to bring in equal access to pooling mechanisms such as social health insurance, for example in Kenya, which however still has distinct risk pools at present, with refugees having access to UNHCR support in rural areas. Pakistan is the outlier, with no pooling of risks, though refugees can access subsidised public primary care. In Mauritania, both hosts and refugee suffer from a system in which risk‐pooling is minimal across the board, with continuing high reliance on out‐of‐pocket expenditure (OOPE) outside of services supported by UNHCR for vulnerable refugees. There may be a future possibility to join a nascent national health insurance, but this has yet to be elaborated.

##### 3.2.2.3 Purchasing

A similar spectrum exists for purchasing arrangements, ranging from distinct (unintegrated) systems in Pakistan to fully integrated in KRI, Peru and Zambia. Kenya is operating a hybrid system, with some harmonised purchasing but parallel systems continuing to function, especially in rural areas, while in Mauritania, purchasing is not integrated in general but there are important programmes like the World Bank‐funded INAYA which use a performance‐based financing (PBF) approach that benefits PHC facilities in and out of camps in the rural areas.

It is important to note that while purchasing may be integrated in some of the countries, the issue of resource allocation taking into account the refugee populations in particular areas was raised in KRI and Peru. Both highlighted problems flowing from the fact that public fund allocations for health care have not been adjusted to reflect the additional refugee populations in each area.

##### 3.2.2.4 Benefits Packages

Entitlements to benefits packages are equal for host and refugee populations in KRI (free primary and subsidised secondary care), as well as in Peru and Zambia, though refugees often face more indirect barriers to benefiting from entitlements (such as low levels of awareness of entitlements to the health insurance programme in Peru, or challenges accessing wider family and social support to effectively access care in Zambia). Given gaps in public provision, OOPE often have to be made for out‐of‐stock medicines, additional procedures or co‐payments for costly referrals (e.g., in KRI), but this is a shared burden with the host population. Some additional support for refugees with specific challenges such as chronic illnesses and mental health conditions are provided on an individual basis in settings such as Zambia, with UNHCR support.

In Kenya, access to primary care is equal across the two groups, however, refugees can access support for secondary care if they are considered vulnerable and need to access priority or emergency services. In Mauritania, refugees had better access until recently than host populations, as they were exempted from fees for services in camp facilities and in accredited urban health facilities, however, this is now changing with reductions in aid and the introduction of fees (a process of levelling down, in effect). In Pakistan, refugees share access to basic free public health services with wider citizens, but lack eligibility for social safety nets like the Sehat card and Benazir Income Support Programme (BISP) or health insurance schemes which would provide protection from OOPE at secondary level and beyond.

#### 3.2.3 Enablers and Barriers

As noted above, the increasing use of development funding, especially from the World Bank, has been an enabler of integration in settings such as Kenya and Mauritania in that funds are deployed to strengthen public systems used by both communities, which may also prove more sustainable and more supportive of social cohesion in not differentiating by status. Universalist configuration of the health system, such as having a free package of primary care for all Zambians and Kenyans which could be expanded to include refugees, also emerges as an enabler, as does the growth in pooling mechanisms such as social health insurance, which enable refugees to join a shared risk pool, either supported by domestic public funds (e.g., in Peru) or, as a transitional arrangement, by international aid (e.g., from UNHCR for vulnerable households in Kenya). Refugee households may need additional support to access these systems, however, as noted in Peru for unregistered households, for example.

Where national public financial management systems start to incorporate refugees in their fund allocation and budgeting, this is a very important enabler of adequate provision in those areas, and our case studies highlight some positive examples where this is occurring (e.g., public funding provided through the INAYA project for Mbera refugee camp in Mauritania) or planned (e.g., in the Kalobeyei Integrated Socio‐economic Development Plan in Kenya), or is lacking (e.g., in KRI and Peru). Payment mechanisms where payments follow individuals or utilisation, such as capitation or PBF can also be helpful in ensuring that the arrival of new populations in need, such as refugees, do not overload the local system and that some quality of services can be maintained, as highlighted in the Mauritania case study.

There are however a number of important barriers raised across these settings too, including concerns about the inadequacy of funding to support access to services for vulnerable refugee groups in the current constrained aid climate. Even countries with integrated approaches such as KRI need medium‐term support in managing the substantial additional needs of their refugee population.

The poor functioning of some public risk pooling schemes like SHI is also a concern, in as much as refugees and host communities alike suffer from delayed payments to providers, with knock‐on effects on demands for OOPE from users, which undermine the intended benefits of insurance in settings like Kenya. In the longer term, to move away from dependence and enable contributions, supporting rights of refugees and migrants to work is important in all settings. In the short term, equalising funding flows to ensure greater horizontal equity is also important. For example, in some settings such as Mauritania, refugees outside camps and host populations face higher cost sharing compared to refugees in camp settlements, which need to be gradually equalised, ideally by reducing OOPE for the disadvantaged non‐camp population, if possible.

### 3.3 Service Delivery

#### 3.3.1 Attributes of Service Delivery Integration

For service delivery, integration includes refugees and host populations having access to the same services and using the same care pathways (adjusted for their needs), including for primary and secondary or referral care. Within this block, we have also included consideration of whether refugee health staff are able to work in host country health facilities (and on what basis).

#### 3.3.2 Patterns in Case Study Countries

Although all systems started with a period of parallel provision, pathways for primary health care seeking by refugees are now broadly the same as for host communities in most of the countries, with all communities also having equal access to public health programmes. Exceptions for primary care are Mauritania and Kenya, where refugees in camps are still able to access higher quality care as a legacy of humanitarian investments in facilities and staff. Service delivery is more integrated in urban areas of Mauritania and Kenya, with weak local health systems a concern for both communities here.

As highlighted above, shared facilities do not mean that all access barriers are equalised, however. Refugees faced additional financial and administrative barriers to access secondary care in Pakistan, for example, and often have more complex health needs, including mental health, which local public health systems cannot adequately meet. Mental health services, for example, are noted as lacking in some of the case studies, such as Zambia.

In relation to referrals, refugees sometimes benefit from more support than host communities—for example, in Zambia, UNHCR facilitates their access to secondary and tertiary care, also assuring ambulance services for settlement communities and nearby host communities, and in Mauritania referrals and related expenses for camp‐based refugees are also covered. In urban areas, secondary and tertiary care are supported by UNHCR but only with referrals from the primary level. In KRI and Kenya, there remains some international support for some of the referral costs of vulnerable refugees with priority conditions, including medicines.

##### 3.3.2.1 Inclusion of Refugee Health Staff

In relation to allowing qualified refugee staff to work in the local health system, Peru is the most permissive of the country cases, now allowing them to work in the public system if qualifications are validated and tests passed. On the other end of the spectrum is Zambia, where they are not able to be employed.

In Kenya, refugee health staff qualifications are not recognised and they normally work as incentive‐based staff for NGO programmes, though local training pathways are now opening up. A similar situation is found in KRI, where qualification and language barriers prevent formal employment, though staff work in a voluntary capacity, for NGOs or in ad hoc ways. Some, once licenced, practice in private clinics or hospitals or even establish their own clinics, however, few are formally employed as civil servants​. Conditions often involve lower pay or stipends (for volunteers) and sometimes non‐permanent status. Similarly, in Pakistan refugee health staff can work on projects on a contractual basis but are not integrated into the formal workforce. Interestingly, health facility autonomy in rural Mauritania has been an enabler, allowing them to recruit Malian refugees as staff, alongside Mauritanians.

Many countries struggle to staff remote facilities serving refugees, so addressing this issue is in the interests of all parties—strengthening the local health system, improving the situation of the health staff themselves and also reducing language and cultural barriers for the refugee community. However, policies in the case study countries have not yet systematically absorbed refugee health workers into the public workforce, though their contributions are increasingly recognized.

#### 3.3.3 Enablers and Barriers

##### 3.3.3.1 Enablers

A number of enablers are noted in the country case studies, which include key steps taken on the path from parallel to integrated provision. The gradual transition of facilities into public ownership is one of these. This can proceed in stages. For example, in Kenya, all facilities have now been registered with the government, and efforts are underway to align them with the Ministry of Health structure, which will entail the use of MOH tools, including health information system and surveillance, logistics and supply of commodities, coordination, management structures (creation of health management boards for each facility), financing, and social health insurance accreditation. This includes initiatives such as training, assigning master facility numbers (to become registered in the public health system), and supplying drugs through the Kenya Medical Supplies Authority (KEMSA). A coordination platform between health partners in camp and MOH exists, with the sub‐county medical officer for health as co‐chair. However, challenges remain as in practice the management of facilities is still done by NGOs, funding is mostly external and parallel to public financial management systems and payroll has not yet been integrated. Further, while facilities in the camp receive some commodities through the national supply chain system, and specifically commodities related to disease‐specific programmes are supplied through KEMSA, Essential Medicines and Medical Supplies (EMMS) are still supplied by UNHCR directly to health providers in the camps. This process is best undertaken in a gradual and managed way which allows for capacity building, adjustment of policies and systems, and development of awareness by communities. This was highlighted in the KRI, where the first phase deployed co‐management of facilities, with government staff embedded in camp services, followed by investment in public facilities, then closure of parallel facilities to ensure a gradual transition with continuity of care.

Opening up facilities which were previously serving refugees alone to all community members (reverse integration of sorts) is another approach adopted in settings like rural Kenya, along with bringing specialists from higher level public facilities to manage cases in refugee‐serving clinics, in order to reduce costs and increase the accessibility of services.

Quality of care and trust in public services is a key factor in all settings. The importance of providing additional support to public facilities to facilitate the transition was highlighted in Kenya, where participants talked of allocating more supplies and laboratory support to ensure that refugees were not seen as an additional burden. Regulation of dual practice (health workers working privately, alongside public service) and ensuring availability of drugs, supplies and equipment were highlighted as necessary measures in urban Mauritania, possibly supported by the planned national health insurance programme. Allowing facilities more autonomy can also be an enabler, as highlighted in Mauritania, where autonomy combined with PBF resources has enabled quality of service provision to be maintained despite hand‐over to a less well‐resourced public health system.

Continued external support, at least in the medium term, can support the gradual transition and necessary improvements to the public health system, especially when humanitarian funding is receding and the public system has limited capacity, as highlighted in the KRI case study. This period can be a window of opportunity for reforms, such as introducing combined community outreach or telemedicine services, which funders can support.

Committed actors—individuals and groups—which make systems more responsive also emerge as very significant, especially in Peru, where community organisations played an important linking role between communities and systems, while key individuals within the public health system were noted to have ensured responsiveness (which is both a strength and a potential weakness, if systems depend on the energy and motivation of individuals to function well).

In relation to staffing, opening local training options for refugees alongside locals was highlighted as an important enabler of greater inclusion in Kenya, with UNHCR support, and in Pakistan, where it has helped to expand access to midwives.

##### 3.3.3.2 Barriers

In relation to barriers for integration of service delivery, differential quality of the starting parallel systems emerged as one of the challenges on the supply side. For example, in Mauritania, disparities in service availability, quality and staffing inside and outside of refugee camps need to be reduced in order to ensure alignment and as part of integration. Underlying health system weaknesses also need to be addressed—for example, in Kenya, UNHCR procurement of medicines and supplies through the public supply chain (KEMSA) requires improvements to quality assurance, which are being addressed. In Zambia, unattractive conditions for staff to work in settlement clinics were highlighted, with limited resources, power shortages and inadequate staff accommodation reducing the willingness of professionals to be employed at settlement health facilities (which contributes to understaffing, high workloads and poor quality of care).

Many of the barriers were reported to lie on the community side, however. Across all case studies, issues of documentation arise as barriers for access to services for refugees, and especially for secondary care and in urban areas. In Nairobi, Kenya, for example, refugees without authorisation to live in Nairobi reported avoiding public facilities because of fear of arrest, contributing to worse health access than the host population. Refugees in Nouakchott, Mauritania, also reported that getting refugee status in urban areas was harder than in the camp. Afghans lacking Proof of Registration (PoR) and Afghan Citizenship (ACC) cards may face service denials in Pakistan. In Peru, not all refugees and migrants have the necessary Foreigners' ID due to restrictions in regularisation pathways, and health and administrative staff often lack knowledge about migrant health regulations. In Zambia, participants noted that some health workers confuse refugees with economic migrants, resulting in unfriendly attitudes towards them and also in refugees being asked to pay for care, while the enhanced documentation needed by refugees for referrals or emergency access (mobility passes) can create added barriers to accessing care.

Language barriers are also a significant factor. For example, in Mauritania, GMB participants spoke of language barriers being common and said that accompaniment is often required for health facility visits. Lack of awareness by users and knowledge about how to access the health system was highlighted in settings like Peru, where participants highlighted the complexity of accessing health services, particularly for forcibly displaced individuals without support networks in the country.

Wider factors beyond the sector also play a role—for example, in Peru, participants reported that current policies were influenced by a securitization outlook, and that this promoted leaving in place barriers to service access for refugees, including barriers to documentation, which is exacerbated by growing xenophobia, although attitudes had been relaxed somewhat during COVID‐19.

On the refugee health worker side, as highlighted, non‐recognition of home country qualifications is a major barrier to inclusion in the health workforce, which also affects community trust in the health system and language barriers. Some countries reported this to be an area which government and partners are working to address—for example assessing equivalence so that refugees can take up roles in the health system in Kenya. In others, pathways to equalisation are emerging for some cadres but not others—for example, Syrian doctors can pass exams to have their certificates recognised in KRI, but nurses and other medical staff do not have the same options. Language issues are also a barrier for staff (e.g., Syrian health staff are used to working in Arabic, while medical staff train in the English language in KRI).

### 3.4 Health Information Systems

#### 3.4.1 Attributes of Health Information System Integration

Integration here is considered primarily in relation to health information systems and surveys and whether they provide equally granular data on both populations, collected through shared tools and systems.

#### 3.4.2 Patterns in Case Study Countries

All of the case study countries started with parallel health information systems in the emergency phase and some retain parallel components, such as donor or NGO‐supported surveys of refugee health utilisation and disease incidence. However, increasingly health information systems are being integrated between refugee‐service facilities and the wider public health system, using the DHIS or equivalent, which improves efficiency and sustainability but in all of our case study countries, refugee status identification has been lost in the process. This is sometimes justified as avoidance of stigma; however, it does create the major challenges of losing the ability to plan for and meet specific refugee health needs.

Information systems used by the different stakeholders, such as NGOs but also WHO, UNHCR and the MoH were noted to be unaligned, for example in KRI, where information system fragmentation is raised, alongside underuse of existing data on refugees (which is adequate at primary level but missing in hospital data). Where information continues to be collected on the refugee populations specifically, such as on consultations for reimbursement purposes in Mauritania, it is not analysed further or linked to other datasets, such as coverage, partly due to uncertainties about changing population denominators. Information system fragmentation and failure to use data for resource allocation or decision‐making is also highlighted in Peru.

These features—limited disaggregation in public health information systems, fragmentation of information systems operated by other stakeholders, and lack of use made of refugee health data for planning purposes—mean that for research purposes, such as this study, very limited secondary data was available to answer important questions, such as on relative health care coverage, financial protection, costs and efficiency.

#### 3.4.3 Enablers and Barriers

We found mainly barriers in this domain, although some of those have been overcome. One practical challenge was systems which are not interoperable: for example, when Kenya moved from the National Health Insurance Fund to the Social Health Insurance Fund, it was configured for national ID entry, which did not match to refugee ID characters. UNHCR had to work with partners to ensure that refugee documents are now recognised and accepted by the system.

More of an obstacle was resistance from refugees, who feared that being integrated into Kenyan national population systems for means testing (to determine monthly premia for SHI) would make their residence permanent, reducing chances of resettlement in a third country. More broadly, participants reported that lack of trust and fears related to lack of documentation hamper the inclusion of refugees in surveys, especially in urban areas, where their response rate remains low and questions about refugee and documentation status are too sensitive to be asked in survey questionnaires.

### 3.5 Outcomes and Implications of Health System Integration

Secondary data limitations affect our ability to tie health system integration to outcomes such as coverage, quality of care, financial protection, efficiency, equity and health outcomes in a robust way and the linkages with social cohesion are described in another paper [9]. However, the case studies do shed light on some health systems effects and interlinkages. As expected, the outcomes of refugee inclusion and health system integration are noted to depend crucially on the state of the wider health system. For example, in Mauritania, the weak capacity in terms of governance and leadership of the Mauritanian MoH, the hierarchical nature of the administrative system and its limited decentralisation, along with dependence on external aid and limited domestic prioritisation of health were all perceived to limit the ability of the health system to respond to dynamic challenges affecting host and refugee populations. Equally, an overstretched health system was highlighted in the Peru case study, which has a comprehensive package of care on offer at primary, secondary and tertiary levels, but which is seen as unsustainable, overly curative in focus and lacking prioritisation mechanisms. All case studies highlight service gaps and resource constraints in local health care systems which impact refugees and host communities alike.

As a result in part of the different starting points and contexts, documented outcomes of integration varied across the settings. In some, integration was reported to lead to reduced costs of seeking care and increased access for refugees. For example, in urban settings in Kenya, refugees were previously required to move relatively far to seek care at specific facilities, which added to their transport costs and also increased risks of detention along the way. Integration of healthcare provision aimed to make services available closer to the refugees' residences.

In other settings, however, financial protection can worsen, especially when refugees previously accessed care, free of cost, at better supported facilities. This was the case in rural Mauritania, for example, where fee exemptions were in place for all patients, regardless of their vulnerability or income, and for all levels of care, and high‐quality care was available. In contrast, host communities faced high OOPE with few exemptions in place except for preventative services and deliveries. In urban areas, the costs of care for refugees were covered by UNHCR through its implementing partners. As such, refugees should have been protected from incurring high costs for healthcare. However, the low quality of public facilities, lack of drugs, complex gatekeeping system and other barriers to access meant that in many cases urban refugees sought care elsewhere, facing the same high costs as their host community. This is in line with the spirit of inclusion and parity with nationals, but refugees often face other barriers to economic and financial inclusion compared to nationals.

Equally, a switch from parallel provision can, at least in the short term, reduce the level of health care access and quality of care that they enjoyed. In Kenya, for example, many respondents suggested that the coverage and accessibility of services was better for refugees in the focal camps, compared to the host population, due to better health worker to patient ratios and support for referrals, as also highlighted in rural Mauritania. In KRI, the shift from specific services for camp‐based refugees to integrated public services, while offering positive features of inclusion, has put an increased strain on available services and led to the loss of tailored interventions previously delivered within camps. Refugees now face similar challenges to host communities, in terms of more fragmented care pathways, longer wait times for referrals and diagnostics at primary health care in large communities and secondary healthcare, and greater geographical and financial barriers (especially for those in remote camps). This is worsened when there is discrimination or lack of clarity by health staff on the entitlements of refugees, as discussed above, or language barriers which are not addressed (e.g., through lack of provision of qualified interpreters in facilities, leading to inadequate care).

## 4 Discussion

In this paper, we have drawn together evidence from six country case studies on how different components of health systems—especially the governance, health financing, service delivery and health information systems—have responded to the refugee inclusion agenda as countries transition from emergency to settled phases, what has enabled those responses and what their implications are. While acknowledging the very different contexts and drivers, which are discussed in a companion piece [8], we aimed to understand patterns and lessons.

In Figure 2, we draw together some of the key factors which have emerged as important for refugee inclusion in national health systems from our study. Some of the factors are hard to influence, such as the numbers of refugees arriving, however these variables are important in influencing the strategies for strengthening integration which may be feasible or relevant. Most importantly, however, the figure indicates recurrent leverage points that have emerged from our analyses, which indicate issues on which policymakers can focus to improve outcomes of inclusion of refugees in national health systems. These include the importance of strengthening local health systems (and their sub‐systems, such as health information, public financial management and procurement) and ensuring that the additional services for refugees do not cause quality of care to fall for hosts and refugees. Underlying that is adequate domestic prioritisation of health care and improved donor coordination to support effective risk pooling, which is a key entry point for reforms. Enabling refugee health workers (not just doctors, but wider health professionals too) to join the local health workforce is another priority which can be beneficial in multiple ways, including economic empowerment of refugees, addressing health workers shortages (or maldistribution) and reducing cultural and language barriers for refugee communities.

**FIGURE 2:**
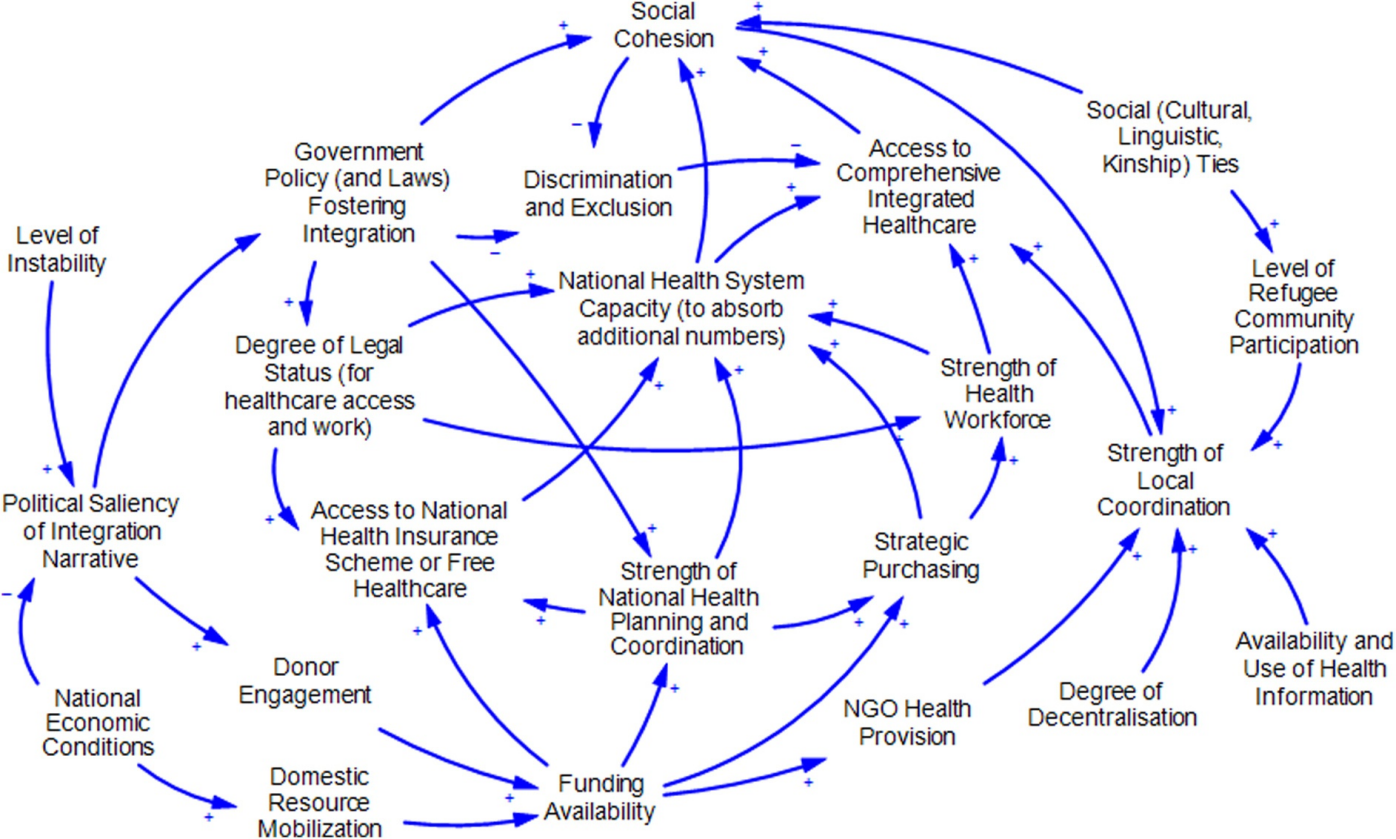
Factors affecting health care integration in six case study countries.

Inclusive policies and positive framing which aims to reduce stigma and highlight refugee contributions also emerged as an enabler in contexts like KRI of smooth integration and benefits for all communities. Effective decentralisation and strengthened leadership in the health sector, as well as community engagement and refugee consultation, also emerge as potential leverage points to support the integration process and ensure that it is well adapted to different contexts. We note, for example, that rural and urban refugees often face different challenges and that integration may require different measures to ensure their inclusion in risk pooling mechanisms and in making local health systems more responsive. Better and harmonised collection of routine data and effective disaggregated data analysis for policy‐making are also essential to be able to monitor progress and inform strategic decisions towards health system integration. Case studies also documented how social cohesion could both be strengthened through equitable access to comprehensive healthcare and itself be of value—in combatting discrimination and exclusion and strengthening local participation—in further enhancing such access. We explore this interaction in more depth elsewhere [9].

From an external funder perspective, there are also lessons in relation to supporting a gradual and well‐coordinated transition, in which funding and responsibility is gradually transferred to public systems, including facility management, procurement, and health worker pay, while these systems are reinforced.

While integration is the normative direction of travel globally, and is important locally to be able to sustain services for refugees over time (now more than ever with global aid cuts [19]), it is clear from these case studies that many challenges remain and some domains, such as refugee inclusion in decision‐making and making effective use of information systems, were scored relatively poorly across the board. Better outcomes for refugees and hosts will not follow unless these enablers and leverage points are addressed. There were inter‐linkages evident, with certain countries, like KRI, Peru and Zambia performing better across the health system domains, while others, notably Pakistan, performed less well across them. This reflects some of the drivers which are external to the sector, such as the current hostile political context and repatriation of Afghan refugees in Pakistan, which we analyse in a separate paper [8], as well as the interconnections between health system domains. In addition to context factors, contingent events such as shocks can play a role in shifting policy. For example, in a number of case studies, such as KRI, the COVID‐19 pandemic contributed to further inclusion. Similarly, in Peru, it triggered the expansion of insurance to all those not previously covered, including refugees and migrants with regular status.

Patterns of integration varied between primary and secondary care: as parallel structures are more commonly established for primary care in emergency phases, the transition involves a gradual shift of management and ownership or transfer to local public facilities for primary care, whereas for secondary care, the shift is more between external funders reimbursing individual services and switching to broader system support.

Inclusion of refugees in national health systems also implies an acknowledgement of the need for national health system transformation or strengthening to achieve equitable health outcomes. For example, refugee populations are likely to experience higher mental health needs, thus requiring support for such conditions. In Peru, this has been prioritised, but instead of making these services available only for refugees, mental health care has been extended to also include local populations.

Many of our findings cohere with wider studies [7]. For example, we note the importance of risk pooling as an entry point and the advantage of building on existing mechanisms, such as SHI, though also that it is not a panacea, as many barriers to joining these schemes exist and coverage and effective protection are often low [4]. We also highlight the importance of the shift from humanitarian to development funding, which can provide a more stable, predictable co‐financing source, for example in settings where World Bank funding has supported health systems serving both communities. Integrating revenue streams is perhaps the least urgent of the health financing domains—if funding comes from separate sources but is used to cover host and refugee populations with harmonised benefits packages and purchasing mechanisms, then the most important dimensions of health financing integration may have been achieved.

However, the importance of wider non‐financial barriers to coverage is also very clear—addressing information gaps, misunderstandings by staff on entitlements, fear of detention and stigma are all key to making refugee inclusion effective. This concords with a recent related review, which highlights that expanding work rights, harmonizing schemes, providing clear eligibility rules, simplifying enrolment, providing protection from immigration authorities and addressing means‐testing complexities are all key to equitable, sustainable health financing for mobile populations [20].

We find that primary care services are more commonly equalised and integrated for refugees and host communities than secondary care, due to the latter's higher cost and unpredictability [5]. From the literature review conducted before the case studies, only Uganda had fully equalised services for all levels of health care, also adjusting its resource allocation formula to reflect refugee numbers living in different areas [7]. The challenges noted on health information systems are also highlighted in recent studies of this topic [4, 21, 22], though it is interesting that some countries have managed to keep disaggregated information on refugees while pursuing integrated health information systems (the positive example of Colombia is cited in the World Bank study).

As highlighted in the health information section, we faced major limitations in accessing secondary datasets which could help us to develop insights on changing costs of care under integration and impacts of integration on efficiency and equity. This should be a high priority for future research. We also only sampled 2‐3 sites per country, focusing on specific refugee populations, and so cannot generalise findings beyond these contexts. A final main limitation to acknowledge is that the global context is very changeable at the moment and findings from the period of research (2023 to early 2025 for country data collection) may no longer reflect the current situation on the ground. In particular, budget cuts affecting UNHCR and other actors is impacting on funding for refugees in the case study countries, as well as technical support at country level.

## 5 Conclusion

Our analysis across specific contexts in six low and middle‐income countries highlights that inclusion of refugees into national health systems is complex, non‐binary and comprises of multiple elements and pathways which are inter‐connected but distinct, with choices and trade‐offs to be made. In the short term, system integration may result in a reduction in access, quality and financial protection for refugees, especially where they benefited from a higher level of resources in the context of a strained host health system. However, committed government leadership, coordinated international support, and considered investments in strengthening key components of the local health system—including enabling refugee health staff to engage effectively in it, strengthening risk pooling and public financial management systems, supporting holistic services packages and improving the data systems which are needed for tracking and decision‐making—can make the transition one which benefits both communities.

## Funding

Funding for this research was granted by the United Nation's High Commissioner for Refugees (UNHCR), under award 2022_RFP_002.

## Conflicts of Interest

The authors declare no conflicts of interest.

## Supporting information


Supporting Information S1

Supporting Information S2

## Data Availability

The data that support the findings of this study are available from the corresponding author upon reasonable request.
