# Supplementary material for: Inclusion of Refugees Into National Health Systems: Enablers, Barriers and Lessons From Six Country Case Studies
Source: Int J Health Plann Manage. 2026 Jul 23;41(6):1146–58. doi: 10.1002/hpm.70109 (PMC13730255; doi:10.1002/hpm.70109)
Supplement: Supplementary file 1 [file HPM-41-1146-s001.docx]

|  | **Kenya** | **KRI** | **Mauritania** | **Pakistan** | | **Peru** | **Zambia** |
| --- | --- | --- | --- | --- | --- | --- | --- |
| Refugee population overview (see (8) for more details | 836,905 in Feb 2025, mainly from Somalia (54%), South Sudan (32%), DRC (7%) and others (7%) | 270,000 in Dec 2024; 90% Syrians, mainly Kurds | 175,332 in March 2025; 95% from Mali | 1,363,948 in May 2025, almost all from Afghanistan | | 1.66 million Venezuelan refugees and migrants in June 2025, including 530,369 asylum seekers and 4975 recognised refugees. | 110,518 in April 2025, from DRC (78%), Burundi (14%); and others (8%) |
| **Governance** | | | | | | | |
| Integrated planning for hosts and refugees (e.g. in national strategies and policies) | There are legislative acts and frameworks that guide refugee integration from a whole of society perspective (not only health specific), including the Refugee Act of 2021 and the Shirika Plan, and at county level the Integrated Social and Economic Development Programmes, which are an integral part of the County Integrated Development Plans.  The 2021 Act was designed to strengthen partnerships between the government and NGOs, fostering a more integrated approach to healthcare delivery and supporting refugees' transition from humanitarian support to an integrated approach to service delivery.  The implementation of the 2021 Refugee Act and of the Shirika Plan is only starting. Similarly at county level, road maps and Development programmes are not yet fully implemented. However, progress has been made for example in administrative integration with camps being designed as municipalities.  In the health sector, policies and reforms often overlook refugee-specific issues (e.g. their inclusion in the ongoing social health insurance reforms). | Refugees included in national system after initial emergency phase, but without legislative framework. Integrated planning for hosts and refugees (e.g. in national strategies and policies). There is no specific refugee law in Iraq, but the KRI has adopted inclusive governance measures allowing refugees equal access to public services, including health. Early in the crisis, the KRG allocated domestic budgets for refugee health and infrastructure before donor engagement. Over time, refugee health was integrated into regular MoH structures through coordination with UNHCR, WHO, and DoHs. Policies are ad hoc rather than legislative, but supported by frameworks such as the Regional Human Rights Plan, and National Migration Strategy (aligned with GCR and Iraq Vision 2030), which includes refugee inclusion goals. Integration is implemented through administrative practice - closure of camp PHCs and transfer of services to nearby public PHCs- rather than formal law. | In Mauritania, roadmap for integration in the healthcare sector and in other sectors, but not full integration as yet in national plans. Refugees not mentioned in national plans, e.g. health financing strategy.    Ownership and management of health facilities in camp transferred to public health authorities and camp facilities included in health system mapping and plans. The process was helped by the operational and financial autonomy of facilities, granted through the PBF scheme (see financing below) which allowed them to continue receiving funding and managing it independently. | Provincial health departments are responsible for policymaking, service delivery and regulation, while UNHCR, WHO, and NGOs provide funding, technical support, and service delivery support. However, efforts remain fragmented, project-based approach, and short-term, with delayed MoUs and limited coordination. No integrated national or provincial framework links refugee and host health systems. | | Many guidelines do not explicitly list refugees and migrants, which affects budgets and services delivered (though efforts are made to change that in certain areas such as vaccinations, HIV and TB). | Integrated approach led by Ministry of Home Affairs and Internal Security, which also ensures coordination with the Office of the Commissioner for Refugees and Ministry of Health. Refugees and migrants are specifically mentioned within national plans and policies, with health strategies detailed similar to other marginalised groups. New policy in 2024 specifically noting integration as requirement in diverse sectors – including health, education, social protection, rights to bank-accounts, settling outside of camps and purchasing land are also included. |
| Refugee inclusion (representation and voice) | The Refugee Act (2021) calls for the inclusion of refugees in national and county development plans. The Shirika plan (2023) builds on this by promoting a whole-of-government, whole-of-society approach that embeds refugee participation as key to integrated planning. Refugees have also been included and engaged in the development of KISEDEP /County integrated Development Plans.    The MoU (2023) between UNHCR and Turkana County explicitly recognizes refugee participation in facility oversight and management discussions | Refugees represented in health governance through standard channels, in patient satisfaction surveys or community boards | In line with national policies, community governance has been strengthened in the camp facilities with the establishment of Health Committees (Comités de Santé – COSA), which did not exist in previous phases. COSAs have been created in order to bolster coordination between health service providers and the refugee community, and appear to be well functioning.  It was also noted that the multiple and active community organisations in Mbera camp are a driver of change.  Urban refugees more overlooked as scattered in population | Refugees do not have formal representation in health planning, policymaking, or coordination forums. | | Refugee- and migrant-led organisations are implementing partners, both at the emergency and protracted stage. They are included in the local cross-sectoral coordination structures and act as a communication channel between the refugee and migrant communities and IOs and NGOs, as well as providing specific health and other services. | Historically, this has not been a priority, however, refugees demanded greater involvement in planning and assessment. The 2024 Refugee Policy, where refugee participation was called for in all the fora where decisions are made affecting them has passed and the situation is now more inclusive of refugee leadership and representation. |
| Effective coordination of humanitarian and development actors | At the highest levels, the two actors that are central for the coordination of policies and strategies are UNHCR and the DRS, also in partnership with county governments, ensuring good coordination at each level. There are 62 partners working on refugee issues in different sectors and with differing roles in and around the camps, which make coordination extremely challenging. | During the early years of the refugee crisis, healthcare services for refugees were managed through a multi-stakeholder approach involving the General Directorate of Health, UNHCR, WHO, UNFPA, and UNICEF. Later this merged into routine sector management processes, e.g. Health Sector Coordination Meetings and Humanitarian Cluster. With funding reductions from international agencies, the DoH took over the management of healthcare for both locals and refugees, providing coordination at regional level, with technical and financial support from UN agencies and NGOs | A *Groupe Thematique Santé et Refugies* (GTSR) has been established at national level, with the participation of all relevant actors including government, funders and implementing partners. However, such coordination mechanisms are not yet implemented at regional and district level, and the focus is more on operational than strategic matters. | Coordination among stakeholders takes place through inter-agency working groups, task forces, and coordination committees, but these mechanisms remain weak and inconsistent. Collaboration is affected by bureaucratic delays, overlapping mandates, and separate meetings held by the Afghan Commissionerate, health departments, and NGOs. | | Functional Unit for the Health of Migrant and Border Populations established in 2020 in MoH but lacks permanent budget and staffing and exists in parallel to regular planning functions. One of the principal spaces for coordination is the Working Group for Refugees and Migrants (Grupo de Trabajo para Refugiados y Migrantes, GTRM). The health area of the sub-group is co-led by MINSA and WHO regional office. | The Office of the Commissioner of Refugees (COR), established in 2017, supports the coordination of ministries working with other partners active in refugee related programmes, including MoH and UNHCR. In health, KI highlighted health coordination committees and mechanisms at various levels (district, provincial, national) to ensure effective communication, planning, and collaboration among stakeholders involved in the refugee response. |
| **Financing** | | | | | | | |
| Revenue raising – sources drawn from shared sources | Refugee health care funded from multilateral, bilateral and INGO support, but with some support also from the county governments through the Health Facility Improvement Act, which provides additional financial support to facilities.  Development funding, including grants and loans, is playing an increasingly relevant role in supporting healthcare for refugees and host communities and the integration process. In particular, the World Bank launched in early 2025 a 215m USD project called “Building resilient and Responsive Health Systems” (BREHS), which includes 40m USD for Garissa and Turkana counties sourced from the IDA20 Window for Host Communities and Refugees (WHR). It is channelled through public financial systems. | Funding sources now integrated as government is the primary source for hosts and refugees. Initially (2012–2014), health financing for refugees was entirely donor-driven, with UNHCR, WHO, and NGOs covering all costs. From 2015 onwards, KRG gradually assumed responsibility for primary care financing, although domestic contributions remain limited and unpredictable due to fiscal crises. By 2023–24, funding sharply decreased, and most operational costs rely on UNHCR and WHO bridging funds, alongside small DoH budget allocations. Refugees are not yet included in Iraq’s national health budget, and health staff salaries face delays due to KRG financial instability. | Funding decreasing overtime since humanitarian actors have left (especially MSF). In 2023 (last year available), most funding (1m USD) came from UNHCR directly to facilities. Another 900k USD was challenged to facilities through INAYA/PBF scheme. Facilities also receive government funding through budget allocations, but it is minimal (8k USD). Other contributions from external partners in kind and cash also existed. Overall, facilities in camp remain much better. funded than those outside.    Inclusion in the INAYA/PBF programme allowed for the opening of a new funding stream to the health facilities in the camp through the roll-out to camps of an already-existing, public stream, broadly in line with what is available for all public facilities in the pilot regions. | | Some access to public facilities but in general separate funding streams from external funders and UNHCR for refugees. UNHCR provides cash assistance in collaboration with the Commissionerate for Afghan Refugees, aiming to support refugee communities’ health needs, including for medical emergencies. | In the initial phases of the response, UNHCR and IOM supported health service delivery via direct payment. They paid implementing partners directly for health service delivery, and/or ensured that refugees and migrants received cash transfers to enable this. This payment was unconditional and unrestricted with payment directly made to qualifying individuals. In later phases (2024) of the humanitarian response, mechanisms were sought to give people access to immigration regularization by obtaining a Foreigner's Card to join the National Health Insurance (public and full coverage). | Initially funding from UNHCR sources but gradually transitioned to government. The national government is responsible for financing health care, but UNHCR funding used to support health facilities with medicines and equipment, including ambulances in camp areas. The aim is for refugees to gradually contribute towards revenue raising via the National Health Insurance Scheme. |
| Pooling - refugees and host populations sharing risks, being included in the same protection schemes and cross-subsidising | UNHCR act as fund-holder for about 50% of total funding to refugee camps. Other funding is disbursed directly to local partners and providers.  Access to social health insurance represents the main public fund pooling mechanism, pooling funds across refugee and non-refugees. However, many challenges remain. Refugees could register into the National Health Insurance Fund (NHIF) with own funds and with support from UNHCR for the most vulnerable. Significant step though numbers remain relatively low).    Kenya policy changed in 2024 under the UHC Act 2023 and SHIF Act 2024 with a transition from NHIF to SHIF. SHIF is designed to be universal and inclusive meaning it covers all persons who are resident in Kenya. Its operationalisation has been highly contested. Some IT challenges to include refugees, but now overcome. | The KRI operates a non-contributory health insurance programme aiming at UHC, but coverage for refugees remains partial and inconsistent. Households (refugees and hosts) continue to face significant out-of-pocket expenses, especially for medicines. Integration of funding streams has begun in practice, as refugees and hosts now access the same PHCs, financed through the same (limited) public budgets. | Pooling of fund across population groups is made difficult by the absence of fund pooling mechanisms in the Mauritanian health system, where most services are accessed through OOPs. Integration would mean increase of OOP and reduced financial protection for refugees – discussions for the introduction of some user fees/co-payments (*ticket moderateur*) were ongoing in early 2024. With budget cut in 2025, this change has been accelerated and is more drastic, with facilities now reported to be charging all patients including refugees, except very vulnerable/new arrivals still covered by UNHCR. Urban refugees pay user fees in public facilities or access free services that UNHCR then reimburses (limited package, only accredited facilities, diminishing numbers of eligible refugees).  Social health insurance schemes (multiple ones) are in discussion to cover formal sector and vulnerable populations, but have not been fully operationalised yet.  PBF is one pooling mechanism as funds are for both nationals and refugees. | | Refugees and host communities do not share risk through common insurance or protection schemes. The national Sehat Card insurance program covers only Pakistani citizens with valid ID cards. | The country has adopted an approach of incorporating refugees into the health system rather than creating parallel services. There is no formal pooling mechanism for refugees only (except those funded by the international organisations). Refugees and host communities share risk through common insurance protection schemes (access depending on having ID card - carnet de extranjería). The SIS is the main mechanism for financing health services. For all those who enrol, i.e., join the SIS, healthcare costs are covered by the Peruvian government. The SIS is granted free of charge to people who have a national identity card or a foreigner's card. No premiums or co-payments are required, as the insurance offers full coverage (100%). | Health care is free at point of care, with pooling ensured across funding from donors and health insurance; the Office of the commissioner for Refugees is responsible for ensuring that refugee funding appropriately supports programmes. Inclusion of refugees into the national health insurance scheme is an ongoing process, however, this would mean pooling would occur via this mechanism. At the time of research, respondents flagged concerns that this would only be possible with greater access to working opportunities for refugees. However, respondents clarified that insurance could also be accessed via the ‘alien’ registration card refugees had access to, and if needed could be supported by the insurer if the population is vulnerable (as is the case for refugees). However, at the time of the research none of these initiatives were implemented and there was low uptake of insurance due to affordability. |
| Purchasing - shared resource allocation mechanisms (such as budget allocations), contracts for services, and possibly payment systems for services covering both population groups | The MOU between Turkana County and UNHCR formally transferred ownership of health facilities to the county government which allows for co-financing. Budget allocation is done under the county health plans and registered under the Kenya Master Health Facility List, enabling allocation of county recurrent budgets. In Kalobeyei, UNHCR funded reimbursements are being harmonized with MOH payment schedules and rates, ensuring comparable service payments for both populations.  However, county budget allocations remain low. Additionally, the procurement of supplies has continued outside of government systems due to their capacity weakness, with the exception of vertical programmes (which include camp facilities for supplies). | Contracts and budget allocations are channelled through DoH, not separate humanitarian agencies. UNHCR no longer directly contracts PHC staff but provides financial support to DoH, which pay staff and procure medicines through government systems. UNHCR and NGOs still occasionally cover costly referrals and provide emergency medical cash assistance, but most purchasing is now done through public procurement systems shared by refugees and hosts. | Minimal budget allocations from government to facilities, PBF scheme in place for strategic purchasing (reimbursing services provided (quantity) with a quality score). Both these schemes cover both hosts and refugees.  UNHCR purchases health services from facilities in camp and urban areas (accredited), through contracts with implementing partner/NGO (fee for service) only for eligible refugees. | | There are no shared purchasing or resource allocation mechanisms that jointly cover refugees and host populations. Government budget allocations and payment systems apply only to citizens, while refugee healthcare depends on donor-funded projects managed through UNHCR and implementing partners.  UNHCR funds are used mainly for infrastructure, equipment, and capacity-building, not for medicines, vaccines, or staff salaries. Donor-supported initiatives, such as the World Bank’s IDA-18 project, provide targeted support to refugee-hosting areas, but these are parallel and project-specific, not integrated within the national budgeting or contracting systems. | The SIS purchases health services directly from health centres and seeks to guarantee access to a package of services established at the national level. | The MoH retains responsibility over the health services and packages that are to be offered at facility levels. While in the past UNHCR did purchase services from implementing partners, this has now transitioned completely to the government and coverage of services is for both refugees and host populations. However, generally, the service package is restricted and budget allocations remain low. UNHCR does offer some additional funding and minor budget allocations for health, e.g. previously offering some supplementation to facilities which are in the refugee settlements, e.g. via emergency vehicles, medications; or by ensuring that referrals are arranged where absolutely needed for refugees specifically. |
| Benefits packages - having the same entitlements and facing similar costs to accessing them across the two groups | Benefit packages are on paper the same for refugees and citizens. The refugee Act (2021), UHC Act, and SHIF Act provide universal health coverage and financial protection to all persons resident in Kenya; this includes refugees. The Shirika plan reinforces this by embedding refugee health within county and national planning frameworks. Under SHIF refugees face similar premiums and copayment structures as citizens. The fact that healthcare at PHC level is free has helped to ensure the alignment of benefit packages. Some discrepancies remain as services coverage is perceived as better in camps, and some vulnerable refugees receive support for accessing referrals, but it is a small and diminishing number. | Benefit packages are identical for refugees and nationals at PHC and hospital level. Both groups receive free PHC services and subsidized hospital care; differences lie mainly in capacity and medicine stock-outs, not entitlement. Refugees occasionally receive partial assistance for referrals or high-cost care from UNHCR, but policy-wise there is no difference in entitlements. | At the time of the research, entitlements were different for refugees in camp and hosts as refugees could access a broad package of services at no costs while facilities outside of camps largely charge user fees. In urban areas, refugees could access services at no costs in accredited facilities. However, the situation is now changing, with user fees being introduced for most services for refugees too. | | Refugees do not have the same health entitlements as host populations. Pakistani citizens access subsidized care through public facilities and the Sehat Card insurance program, but refugees are excluded from these schemes. The refugees can use government health care facilities without any discrimination where the services are free for the citizens.  The Medical Teaching Institutions (MTI) Act 2015 enacted in KP province introduced user fees at public hospitals, increasing costs for both citizens and refugees in the province, but the impact is greater on refugees who lack identification-based subsidies and insurance program entitlement. | When refugees are covered by the national insurance, they receive the same benefits and packages as the host population under the Public Insurance System (SIS). There are also universal packages for all people in the territory, such as care for children under five, pregnancy, and treatment for HIV and tuberculosis. | At the time of the research, both refugee and host populations had the same entitlements and generally faced similar problems in the access of primary care especially. At secondary level, the challenges are compounded for refugees, as they have less social support than locals, e.g. family etc, in addition to facing the cost of seeking care at higher levels of care. UNHCR supports refugees in seeking care at higher level services by providing transport and related costs for in patient care. However, there are budgetary constraints and logistical delays due to high demand. Affordability concerns for UNHCR have meant that there is emphasis on refugees being part of insurance. |
| Service delivery | | | | | | | |
| Refugees and host population having access to the same services using the same primary care pathways (adjusted for their needs) | Refugees and host communities receive free primary healthcare (levels 1-3) in the same county managed facilities.  Service delivery follows the Kenya Essential Package for Health (KEPH) and county clinical protocols, ensuring standardized care for both groups. Facilities are listed under the Kenya Master Health Facility List (KMHFL) and report into the Kenya Health Information System (KHIS2), meaning they follow the same protocols, data tools, and standards as all other public facilities. Community health promoters (CHPs**)** including trained refugees link both communities to these services, ensuring culturally sensitive outreach and follow-up. | Refugees and hosts access the same public PHCs and hospitals. Camp PHCs were closed between 2023-2025, with refugees redirected to nearby public facilities. Primary care includes vaccinations, maternal/child care, chronic disease management, and MHPSS, fully integrated into the KRI health system. | In Mbera camp, service provision was shaped by humanitarian intervention. At the time of the research, refugees had access to better coverage and quality care at no cost. Some changes to align to national health system have been introduced (for example, nutrition services moved to the hospital, outside of camp), but differences remained, especially given the extreme weakness of the local health system. These imbalances in availability, coverage, accessibility and quality of care made integration more difficult (stark trade-off for refugee health outcomes) but also necessary (to avoid tensions and reduced social cohesion). GMB participants stressed the importance of improving coverage and quality and strengthening local health system.  Differences in urban areas were less stark. Refugees access free health services but only at accredited public facilities, perceived of low quality. | | Refugees and host populations generally access the same public healthcare services and facilities, including Basic Health Units (BHUs), District Headquarters Hospitals (DHQs), and tertiary hospitals, though refugees face greater financial and administrative barriers. Both groups benefit from national health programs such as EPI, TB control, HIV, and leishmaniasis services. | No differentiated services have been implemented in the country for refugees and migrants, beyond specific medical exams or medical attention funded by the International Organisations. | Upon their arrival in Zambia, asylum seekers undergo a formal registration process that grants them legal recognition and access to essential services. Challenges in the arrival and administration processes for refugees are widely acknowledged, however the pathways of care are the same as for the local host population once registration is achieved. |
| Refugees and hosts having access to referrals and secondary care through the same pathways | Patients from refugee and host communities follow the same referral pathways and protocols by the County.  However, transport and ambulance services are pooled though UNHCR and implementing partners often subsidize fuel, vehicles, or emergency transfers. A small number of vulnerable refugees (in camps and in urban areas) also receives subsidised secondary care. In some cases, specialist visits have been organised to camp facilities.  Refugees enrolled in NHIF/SHIF have access to the same referral hospitals and benefit packages, including inpatient and surgical care. | Referral pathways are unified: PHCs refer refugees to hospitals using standard national procedures, and ambulance services use the same emergency number for both populations. UNHCR supports transport or co-pays only for vulnerable refugees. Quality challenges - long wait times, medicine shortages -affect both groups. Integration has reduced duplication and improved social cohesion between host and refugee populations | Referrals for refugees were to public facilities (same package and quality of care), but costs (including transport etc) covered by UNHCR for eligible refugees. Same for urban refugees. | | Refugees and host populations use the same referral pathways for secondary care. Patients from both groups are referred from Basic Health Units (BHUs) to District Headquarters Hospitals (DHQs) or tertiary hospitals for advanced treatment. However, while host communities access subsidized services through the Sehat Card, refugees must pay out-of-pocket due to their exclusion from insurance coverage. | No differentiated services have been implemented in the country for refugees and migrants, beyond extra-mural activities with the help of cooperation. What does vary is whether they are covered by public insurance or will have to pay out of pocket. The latter does cause delays in access to care. | Referrals are handled the same way for refugees and host populations; in settlements, UNHCR has in the past supported transport via ambulance for both host and refugee populations. However, in the case of refugees, UNHCR supports referral costs where appropriate. |
| Refugee health staff being able to work in host country health facilities | The Refugee Act (2021) grants refugees the right to work and participate in Kenya’s social and economic life, including public service, subject to regulation. However, in practice there are still barriers to join the health workforce for refugees. Refugee health personnel currently serve mainly as “incentive staff”, a term used by UNHCR to describe trained refugees employed by implementing NGOs within health facilities, as they are legally unable to receive a salary and qualifications are not easily recognised by Kenyan authorities.  The Kenya Medical Training College (KMTC) Turkana campus supported by county and UNHCR now admits both refugees and host students, a major step toward inclusive professional pipelines. | Refugee health professionals have limited access to national payroll due to Iraqi nationality restrictions. Some Syrian doctors and nurses work informally or under NGO contracts in camps or public PHCs.  Licensing and recognition barriers persist; credential equalization is available only for doctors (not nurses or allied staff), and language differences (Arabic vs. English curricula) complicate certification.  Discussions on future accreditation fast-tracks and inclusion of refugees in health workforce training are ongoing, with UNHCR advocating policy reform to address shortages. | With the integration process, facilities in the camp were able to retain Mauritanian staff, who had been recruited by NGOs in previous phases. This increases the quality of care in camp facilities. However, these health workers have *not* been integrated in the civil service/MoH payroll), but have contracts with the Ministry of Finance. Because of autonomy and fund availability, facilities are able to contract both Mauritanian and Malian/refugee health workers. The recruitment of refugee health workforce was mentioned as an important pathway for integration, and also a factor that ensures service delivery and care are appropriate for refugees. Overall, camp facilities have far more and better-quality staff than public facilities outside camps. | | Afghan refugee health workers, particularly midwives and female healthcare providers, are receiving targeted training and capacity-building support to serve within refugee communities. Some trained Lady Health Visitors (LHVs) and midwives from the Afghan refugee community work in refugee-focused health facilities and outreach programs, often under UNFPA-supported initiatives. Their engagement is primarily on a project or community basis, not through formal integration into Pakistan’s public health workforce. | Refugees can work in health facilities if they have a health-related degree and are able to validate their degree in Peru (including the completion of a theoretical-practical course and test). | At the time of the research, refugee health professionals were not allowed to practice. In settlements, Zambian nationals were acting as health care professionals, but this also came with challenges of high attrition. |
| Health information systems | | | | | | | |
| Health information systems and surveys provide equally granular data on both populations, collected through shared tools and systems | UNHCR has historically collected routine data for refugees, with a parallel approach. This separate HIS continues for the time being, with a focus on incidence and service use, for surveillance and planning purposes. At the same time, efforts to integrate HIS are ongoing. All health facilities serving refugees in Kakuma and Kalobeyei are now registered in the Kenya Master Health Facility List (KMHFL) and report routinely through the Kenya Health Information System (KHIS2) - the same platform used by all public facilities nationwide. However, these data are not disaggregated by refugee status. Integration for census and surveys is being support by the World Bank, but challenges remain in terms of technical capacity and also trust, especially in urban areas, which makes it difficult to gather disaggregated data and also to sample the refugee population correctly. | Initially, data were collected through UNHCR’s IRHIS, parallel to Iraq’s DHIS2. Over time, refugee-serving PHCs were added to the national HMIS, enabling routine data entry under DoH oversight.  However, data are not disaggregated by refugee status, limiting monitoring of equity and outcomes. Integration of systems is ongoing, with plans to unify surveillance and reporting tools across all KRI governorates. WHO and UNHCR support capacity building for joint data management and digital health records pilot projects. | Routine HIS based on DHIS2 but electronic only at district level (below district facilities are still reporting on paper forms). Facilities in the camp required to fill in the same forms as all public facilities and send to district. However, information is not disaggregated by refugee status. Same for PBF data, which is used to calculate payments to facilities.  UNHCR collects own data separately for refugee/camp facilities only. | | Pakistan’s District Health Information System (DHIS) and related reporting tools do not disaggregate data by refugee status. While both refugees and host populations use the same public health facilities, the health information systems lack a specific column or indicator to record refugee data separately. Only EPI and polio programs collect integrated data from all children, regardless of nationality. | The information system is fragmented, and most of them do not have variables that identify whether the person is a national or a foreigner or their country of origin, and those that could have this variable do not identify immigration status. This poses a significant challenge for planning and resource allocation, because the needs and volume of refugees accessing health services are not adequately taken into account. | There are clear data asymmetries between the populations. There are two information systems in place that capture population health data: the Integrated Refugee Health Information System used by UNHCR and the District Health Information System 2 (DHIS) used by the Ministry of Health. Challenges arise because recording across the two systems is different (in terms of what is recorded) and because there is a different level of disaggregation between systems. Generally, there is a view that disaggregating data for refugees vs host populations would not promote inclusivity and integration. |
