# Supplementary material for: Inclusion of Refugees Into National Health Systems: Enablers, Barriers and Lessons From Six Country Case Studies
Source: Int J Health Plann Manage. 2026 Jul 23;41(6):1146–58. doi: 10.1002/hpm.70109 (PMC13730255; doi:10.1002/hpm.70109)
Supplement: Supplementary file 2 [file HPM-41-1146-s002.docx]

# Health system resilience, enhancement and refugee responses project

# Case study protocol

# June 2023

# Table of contents

[Summary of research approach	6](#_Toc139024121)

[Background	6](#_Toc139024122)

[Aims and research questions	7](#_Toc139024123)

[Case study methodology	8](#_Toc139024124)

[Conceptual framework	9](#_Toc139024125)

[Overarching case study design	10](#_Toc139024126)

[Document review	11](#_Toc139024127)

[Secondary data analysis	13](#_Toc139024128)

[Key informant interviews	14](#_Toc139024129)

[Group Model Building	16](#_Toc139024130)

[Overall data analysis and write up	20](#_Toc139024131)

[Quality assurance	21](#_Toc139024132)

[Ethical considerations	22](#_Toc139024133)

[Research uptake and dissemination	24](#_Toc139024134)

[Timeline	25](#_Toc139024135)

[Research team	26](#_Toc139024136)

[Annex 1: Participant Information Sheets	27](#_Toc139024137)

[Annex 2: Consent form	33](#_Toc139024182)

[Annex 3: General topic/interview guide for key informant interviews	34](#_Toc139024184)

[Annex 4: Outline of Group model building workshop	37](#_Toc139024185)

## Summary of research approach

In the face of increasingly protracted crises and tightening budgets, the challenge of providing equitable, accessible, and quality healthcare in ways that are sustainable in the longer term is becoming increasingly central to UNHCR’s public health mandate. Approaches to meeting refugees’ health needs in structures parallel to the national (local) health system might lead to challenges in relation to duplication, fragmentation, high costs and inefficiencies and there is a call for greater inclusion of refugees into national health policies, strategies and plans, as well as the integration of refugees in national health systems. Limited studies exist on the evaluation of ways of funding and organising health responses and their wider effects on the host population in different settings. We aim to fill this gap by conducting a total of six country case studies. Each country will include different sites (up to three) with various characteristics of the response to refugees’ health needs. For each country, we will identify partner(s) which may include individual research consultants or research institutions. These case studies build on a literature review already undertaken by the research team.

The case studies will run in two rounds of three case studies each across the duration of the project. We aim to conduct a rapid review of existing documents and data in each country, up to 30 key informant interviews, as well as Group Model Building (GMB) workshops. Analysis and write up will take place for each case study, but also comparatively across the case study countries, with the aim of identifying lessons learned and making recommendations for future planning on the topic of refugee health responses.

## Background

This work was commissioned by UNHCR in the context of the growing number of refugees and forcibly displaced populations worldwide and the advent of the Global Compact on Refugees^[1]^. The Compact outlines a multi-stakeholder approach to better respond to refugee situations to achieve protection and solutions whilst easing the burden on neighbouring communities and promoting self-reliance. In the face of increasingly protracted crises and budget pressures, the challenge of providing equitable, accessible, and quality healthcare in ways that are sustainable in the long term is becoming increasingly central to UNHCR’s public health mandate.

Approaches to meeting refugees’ health needs in structures parallel to the national (local) health system may lead to challenges in relation to duplication, fragmentation, high costs and inefficiencies and there is a call for greater inclusion of refugees into national health policies, strategies and plans, as well as integration of refugees in national health system^[2]^￼^[3]^￼^[4]^￼. However, inclusion of refugees in national health systems is not straightforward. There can be reluctance or resistance on the part of the recipient government, due to numerous factors, especially if funding is not secure and their own health system is, or faces the risk of becoming, overburdened^[5]^. More evidence is needed on the rationale for, and approaches to, achieving better integration and whether these lead to ultimately better health outcomes for refugee and host populations.

As part of this project, a literature review was conducted which informed the development of this next phase of work^[6]^. The literature identified areas that require further investigation, evidence to build on, and pointed to interesting methods or approaches that we can benefit from when conducting the case studies.

Although findings will remain context-specific, we have highlighted key gaps in the literature that can inform the design of our case studies. In particular, there is a lack of systematic analysis and comparative work on what health system integration means in different contexts, when this is desirable and at what level or degree. The case studies will be a vehicle to consider the types of integration that can be adopted in different parts of the health system, and when each type is appropriate. There is opportunity for a systematic focus on lessons on “how” successful integration can be achieved and exploring facilitators and barriers of health system integration. Finally, the broader linkages between integration and (i) resilience of health systems and (ii) sustainability, efficiency and cost-effectiveness, and (iii) social cohesion may be usefully explored. Our case studies will also be well placed to consolidate lessons from existing or ongoing attempts to increase alignment with government systems, and reflect on under what conditions this increases efficiency and equity of service delivery, social cohesion and health system resilience.

## Aims and research questions

To identify lessons on how to provide appropriate, cost-effective, equitable and sustainable services to refugees, across a diverse set of case studies. Cases will represent different socio-political and economic contexts, diverse health systems and also the diversity of responses across acute, post-emergency and protracted emergency phases.

The overall research question that the country case studies address is:

How, why, and how effectively have countries organized provision of health care for refugees, and what lessons can we draw from this, looking across settings?

Addressing this question will include understanding the following aspects:

1. How context variables such as refugee numbers, origin, background and status, interact with country context (including host context) to influence patterns of health provision for refugees and their outcomes
2. How different configurations of service delivery, governance, financing and monitoring and evaluation of health services for refugees interact with one another to impact access, quality, affordability, equity, resilience and sustainability of health care delivery systems for refugees.
3. What can be learned from the configurations observed in 2 on the impact, desirability and feasibility of different kinds of integration (across the system blocks) in terms of refugee and host provision and outcomes.

These questions follow our conceptual framework. The study will draw lessons across the case studies on context-appropriate configurations that maximise desirable outcomes, including coverage, resilience, sustainability, cost-effectiveness, quality, equity and social cohesion, considering not just refugees but also national systems and host communities. Maximising in this context takes into account trade-offs that have to be made, given the scale of needs and limited resources (i.e. there will be no perfect solutions).

## Case study methodology

To achieve this aim, we examine different countries as case studies. Each case study will document and learn from the different approaches to meeting the health needs of refugees to inform planning and response to future and current displacement. All case studies will have a shared methodology and similar format, in order to facilitate comparative analysis across contexts and effective extraction of key lessons learned on cross cutting themes. Comparative case studies have been shown to enhance explanatory power and analytic generalisability. ^[7]^

Each case study will focus on the unique context of the country, and the methodology will therefore be adequately adapted to each country. This will include adaptation and tailoring of some general terminology mentioned in the protocol e.g. “host” or even “refugees” depending on the locally accepted terminology.

### Conceptual framework

In order to structure the data collection and inform our understanding of the topic, we developed a conceptual framework, which is summarized n Figure 1. The conceptual framework was developed iteratively, starting with an initial draft and then refined as the literature review progressed, to provide a common frame for analysis. Further refinements and improvements will continue to be made through the iterative approach described above.

**Figure 1**: Conceptual framework

The conceptual framework highlights the different elements and sub-elements that we found are key to understanding health system responses for refugees. It starts (on the left-hand side) with an understanding of the broader context: this includes the history and phase of the displacement and of government response (including but also beyond the health sector) in the particular setting; the country context (including legal provisions, economic trends, fiscal space, political settlements, state and level of functioning of the host health system); the numbers, demographic and epidemiological profile, legal status of refugees, as well as their origin, setting and distribution; and finally the refugee health needs and access to services and elements linked with broader social determinants of health.

The middle blocks of the framework comprise specific systems elements, and define the health system response and response approaches to healthcare for refugees. These include service provision (which also covers elements pertaining to human resources for health), governance, financing, and monitoring, evaluation and learning (MEL; which includes health information systems).

Finally, the right-hand side of the framework focuses on the UHC outputs and outcomes, with a focus on understanding how these are shaped or defined by the response approaches; the impact on the wider (national or local) health system, including the potential trade-offs of different approaches and the enablers, barriers and drivers of integration; and finally, looking at the impact on social cohesion, reflecting on how health responses might affect the relationship with the host community and peaceful coexistence between communities.

Cutting across our analysis are the concepts of resilience, sustainability, efficiency and cost- effectiveness, which are of particular relevance to the study and, where relevant and possible, are reflected upon across the different elements of the framework.

### Overarching case study design

The overall approach will be a mixed-methods exploratory case study design.

Within each case study, we will follow a retrospective approach looking back at a relevant period of time defined by the key events at country level, and we will also include participatory approaches as detailed below.

Specific site(s) within the country of interest (selected as part of the process with UNHCR) will be chosen collaboratively with UNHCR representatives and local research partners where relevant. It will be important for site selection to reflect the broad differences in the healthcare responses for refugees in each country, which we know can differ substantially – for example, on the basis of the origin, status and location of the refugees (e.g., urban or rural settlements, camp or not in camps, recent or prolonged displacement, etc.). Within those different contexts, we will identify specific communities or exact sites of relevance from a research perspective to ensure some comparability, but also based on feasibility, access, existing knowledge and networks, and other practical considerations. We envisage including between one and two sites (or a maximum of three in some cases) in each country.

Each case study will start with an initial scoping review of key documentation and any secondary data available, which will allow a better understanding and “landscaping” of the context as well as identify key actors and informants. This will be followed by semi-structured qualitative interviews with key informants and analysis of any further data and document emerging from the interviews, and finally GMB participatory workshops (details provided below).

Although this is the broad sequencing of the methodological approach, each data collection component will be iteratively carried out building on the other components. So, for example, the initial document review will be reviewed and augmented with documents suggested during key informant interviews. The final data analysis and write up will bring together all components, comparing and triangulating information from different data sources, as detailed in the data analysis section.

### Document review

A document review will be conducted to explore existing processes and policies in place in terms of arrangements for healthcare provision for refugees, as part of the country’s health system. We aim to focus on the contextual elements noted in Figure 1 above (including stage and history of displacement, how it has been organised, demography and epidemiology of refugee population, distribution, landscaping of external actors (donors and NGOs), legal situation of refugees, including rights to work, other services, etc.), but also covering as much as possible the other elements of the framework. This includes identifying current issues, progress made so far, challenges and opportunities, as well as any other available evidence, research and programmes supporting the health needs of refugees. Special attention will be drawn to the following variable factors: a) phase of the response b) setting and context e.g. rural vs urban etc. and c) equity dimensions suggested by the response. Furthermore, we aim to assess the capacity of the national health system and the impact on the host system and the communities it serves, including in terms of social cohesion. Additionally, we will examine the current available health financing mechanisms, costing and governance arrangements of the health system response. Finally, the document review will draw upon implications of findings on sustainability and resilience of the health system.

Practically, the review will look at government (Ministry of Health and other governmental agencies) documents; UNHCR, other UN agencies and Health Cluster documents; documents of implementing partners; and other key documents to be co-defined with the relevant stakeholders. We anticipate documents to include national laws and policies, government reports, guidelines, strategies, evaluation reports, research studies, and international and regional agreements or conventions that the country has ratified. We will search for these documents on databases (for peer review literature, PubMed and Google Scholar), government and other agencies websites (for policies and government reports, UN reports, etc.), and by directly contacting national stakeholders and UNHCR. We will also ask for any additional relevant documentation during KIIs. We will review legal and policy frameworks. We will examine national laws, regulations, and policies related to healthcare services for refugees.

As part of the document review and situation analysis we will:

i) Construct a timeline of the response to the refugee arrivals by the national systems and different phase and planning of this response
ii) Document service provision – including service packages and pathways/referral mechanisms, as much as possible exploring coverage, equity, accessibility, quality, etc. of health provision.
iii) Assess coordination structures. This may involve examining intergovernmental coordination mechanisms, partnerships between government agencies and humanitarian organizations, and the roles and responsibilities of various stakeholders involved in providing healthcare to refugees.
iv) Explore the funding mechanisms and financial arrangements related to refugee healthcare. This may involve analysing budget allocations, donor funding, health insurance schemes, or any specific financing mechanisms established for refugee health services.
v) Identify monitoring and evaluation mechanisms in place to assess the effectiveness and impact of refugee health programs. This will include data collection systems, indicators used to measure health outcomes, and evaluation reports that provide insights into the performance of the health system for refugees.

*Data analysis approach*

Data will be extracted from the documents identified and analysed thematically according to the elements of the conceptual framework.

### Secondary data analysis

We will work with partners and UNHCR focal points to establish what secondary data is already available, and gather all that relevant secondary data that is available. We will only use anonymised datasets, that are either publicly available or that can be made available for this study (potentially following anonymisation).

Through secondary data analysis and analysis of data availability we aim to address questions such as:

- What data is available on health outcomes and service outputs and use for both refugee and host populations? What does the data analysis suggest?
- Is there a quantification of health needs for refugee and host populations?
- Is there any data in terms of coverage of health services, equity, accessibility (including financial barriers to care) and quality of care for refugee and host populations? What does this data suggest?
- What data are available on costs of care, and expenditure, from health system or societal perspectives for refugee and host population health care? What do these data suggest regarding budget space/availability, funding flows, and cost-effectiveness of services?
- Is there any integration with MoH health information systems? If not, what are the barriers preventing the integration? Is refugee data accessible/ disaggregated in national systems? If yes, describe the process.
- Where and how is data on refugees stored and accessed, how does this inform decision—making? When and how are data used? This includes consideration of data management, sharing, quality assurance and timeliness/reliability.

*Data analysis approach*

Analysing secondary data related to access to healthcare for refugees as well as the response of health systems requires a comprehensive approach to understand the complexities involved. According to the availability of data, we plan to implement an analytical strategy that focuses on understanding magnitudes, trends, differences between groups, testing hypotheses, studying associations, and evaluating potential causal mechanisms, as follows.

First, we will conduct descriptive statistical analysis to identify magnitudes and differences between natural groups (by sex, host/refugee, by site/camp, health provision arrangements, etc.). Second, we will study data longitudinally, observing differences occurring over time. By examining these statistics, we will gain an overview of the data and identify initial patterns or trends in terms of healthcare access for refugees as well as the main characteristics of health systems and how these change over time.  Longitudinal analysis techniques, such as time series analysis, panel data analysis, or growth curve modelling, can be employed to investigate how access to healthcare evolves over time. This analysis may involve examining trends in healthcare utilization, changes in health outcomes, or shifts in the availability and quality of healthcare services.

Third, we will study factors associated with health outputs and outcomes. When possible, and also informed by the qualitative work, we will define models to investigate if and how health provision arrangements in an area are linked to changes in health outcomes and outputs. This step involves exploring associations between independent variables, such as demographic characteristics (age, sex), refugee status (host/refugee), site/camp location, and health provision arrangements (availability of healthcare facilities, healthcare policies), and dependent variables (health outcomes and outputs). Statistical techniques such as correlation analysis can help identify relationships between variables, while regression analysis (linear or logistic) can assess the impact of independent variables on health outcomes and outputs.

Data will be analysed using R or STATA.

More information on data sources for document review and secondary data analysis can be found in Annex 5.

### Key informant interviews

We will conduct (semi-structured) key informant interviews with participants with experience and knowledge on refugee healthcare provision and integration. Although we will work around the principle of saturation^[8]^, we aim to sample about 30 participants for each case study. The sampling strategy will be based on convenience, diversity and purposive sampling approaches to recruit participants from different categories with sufficient variability to capture different perspectives, such as representatives from:

- Ministry of Health, and other relevant ministries and governmental agencies at national or provincial/regional levels and district levels where possible.
- Public and private health service providers including health managers (e.g. DHMTs)
- UNHCR, WHO and other UN agencies (e.g. UNICEF)
- Donor and funding agencies
- Civil Society / NGOs / iNGOs, particularly those involved in the healthcare response and/or implementation
- Community leaders of refugees and/or host communities

We aim to get gender representation, and measures will be taken into account to mitigate the male dominance in certain settings to ensure as close to equal gender representation in the final sample as possible.

*Data collection*

An initial list identifying key informants across all the categories above will be prepared with the support of UNHCR country office as well as local research partners. Additionally, a snowball technique will be adopted by asking every key informant to suggest new ones. During this process, particular care will be taken to ensure that there are no gaps in covering different categories of informants to ensure broadness of views, but also to ensure all those with critical, relevant knowledge are included.

Respondents will be initially contacted via email, telephone or in person to explain the purpose of the study and propose the interview. Those who agree to participating will be asked to read an information sheet (Annex 1) and (if in agreement) offered to sign a consent form (Annex 2), which also specifically and separately asks for consent for recording the interview. Where the interview is done remotely, there will be an option to take verbal consent and record it using an app on the telephone, or an external recorder (separate recorder) as a separate file and transcribe and store this.

The focal researchers for the country case study (either from the local research team with support from the QMU team or the QMU team directly) will conduct the key informant interviews. Interviews may also be conducted by other members of the (QMU and local) team, depending on availability, practicality, proximity and language skills. It is envisaged that the majority of the interviews will be face-to-face but some could be virtual if that is preferred by the respondent or for feasibility reasons. They will be conducted at a time that is convenient for respondents, in the language with which they are most familiar and (in case of face to face) in a private and safe location. Interviews will last approximately one hour. Whether recording is permitted or not, detailed notes will be taken.

A general version of the topic guide for the interviews is in Annex 3. It covers issues such as service provision arrangements, governance of health systems, financing of the response as well as MEL elements. The topic guide will be adapted to each of the contexts to take into account the specificities of the settings as well as the key issues and gaps in information emerging from the document review. It will be translated in the appropriate language for each case study.

*Data management and analysis*

Recordings of the interviews will be transcribed verbatim, or the notes will be written up as transcripts, if consent to recording was not given. Analysis will be completed in the original language if the QMU team can do so, and only relevant quotes will be translated. If this is not the case, the transcripts will be translated into English.

The study team researchers at QMU and locally will jointly manage the data collection process and manage data. Recordings and notes will be stored on password-protected devices (One Drive System), and consent forms will be kept separately from other data and will be linked to the data via a numerical code. Transcripts will be anonymised as soon as possible after transcription and only anonymised data will be used for the analysis and shared between team members.

Interviews will be analysed using the thematic framework approach which facilitates rigorous and transparent analysis and uses both deductive and inductive approaches^[9]^, working from the initial elements of the conceptual framework, but adding codes as needed. Researchers will first familiarize themselves with the contents emerging and create a coding tree based on the framework mentioned above. After coding the transcripts, researchers will meet to discuss emerging themes by codes and sub-codes and prepare narrative summaries of main accounts^[10]^. The findings from the interviews, data analysis and document review will be triangulated and compiled and will further inform the group model building workshops as detailed below.

### Group Model Building

GMB workshops will be convened to complement the KII data collection. GMB is a participatory approach to engage with stakeholders in understanding an issue from a systems perspective. Vennix^[11]^ defines GMB as “a process in which team members exchange their perceptions of a problem and explore questions such as: what exactly is the problem we face? How did the problematic situation originate? What might be its underlying causes? How can the problem be tackled?”. The workshop aims to visually represent the problem of interest and be facilitated through several scripted group exercises which allow for exploration of dynamic linkages between key system and context elements.

In the context of these case studies, the GMB will aim to draw together findings from the different data collection components above (document review and KIIs) and complement these. GMB workshops will be organised in each country as follows:

**Workshop 1 (one per study site, maybe multiple per country)** will bring together both refugee and host community members, where relevant to do so, as well as local health service providers to explore their perceptions of health system responses and provision during different phases of the emergency response. We anticipate conducting one such workshop per site; if there are two sites per country, then this would mean two workshops in total. In these workshops, participants will be asked to reflect on accessibility and affordability of care, the equity of service provision towards different groups, inclusion of refugees into different types of services and also any existing community-health system relationships. The workshops will also aim to elicit how these factors all affect social cohesion.

**Workshop 2 (one per study country)** will bring together key stakeholders who have direct influence and bearing on refugee service provision at country level. For example, we anticipate these to be policy makers or decision makers of the national health system, representatives of UNHCR, WHO and other organisations with a stake in health service delivery, and representatives of civil society, i/NGOs or private entities assisting with health-related refugee service provision. The focus of this workshop will focus on the organisation and coordination of responses towards refugee health service delivery in the different emergency phases, touching on topics such as priority-setting, financing, monitoring and evaluation, accountability, and integration of refugees into national service provision. The workshops will also aim to elicit perceptions of how responses reflect and affect the resilience of existing health systems and will seek to understand the role of integration in this regard.

**Sampling:** The participant list will be adapted per country to ensure that diverse individuals and experiences are represented per site and country. Each GMB workshop will include between 8-16 participants, purposively identified. The sample for each workshop will aim to be age and as far as possible, gender-balanced, ensure representation of diverse segments of the community as well as the organisations, institutions, and groups targeted. Convenience and snowball sampling will also be used, given that each workshop will last one day.

**Recruitment and consent (see annexes for information sheets, consent forms and guides):** Suitable individuals at community level will be identified by the local research team in partnership with UNHCR and local leaders. Potential participants will be approached by the research team by phone or in person (e.g. in some contexts KIIs will be done during site visits, so recruitment of other participants may also be done in person). The researchers will explain the study verbally, and provide participants with a written information and consent sheet. If the participant agrees to participate, written consent will be secured and their contact details will be retained so they may be invited to the workshop. Personal details of participants will only be held until data collection is complete, so as to ensure that participants can be invited to workshops and/or can be called by the researcher in case of clarifications regarding their workshop input. Consent sheets will be linked to the collected data via a numerical code. Participants’ right to withdraw from the study at any time (without any issues) will be respected, and it will be made clear to participants that no identifiable information will be included in any final reports/materials published or discussed from this work. As GMBs include multiple participants from the same community, an initial part of the welcome for the session will also entail guidance around respecting and maintaining confidentiality of all those present.

Eligibility criteria for community members include:

- Participant being over 16 years old (in the case of those aged 16 to 18, written informed parental consent will be gained for their participation);
- Participant identifies as member of refugee or host (non-displaced) community;
- Participants have accessed or attempted to access health care in the last year for either preventive or curative purposes.

Any participants who have taken part in interviews for this study or are under 16 are not eligible to take part.

In case of representatives of organisations, these will be approached similar as per the KIIs. Information about the study will be disseminated directly to the organisations, with invitations followed up via phone. Written consent will be secured from participants, with the rights to anonymity, withdrawal, confidentiality and privacy explained to all participants.

**Incentives**: In some contexts, it may be appropriate to offer incentives to the community members participating in the GMBs. This is because community participants may miss out on remuneration for one full day’s work and this in turn could exacerbate their vulnerability. Offering incentives will be decided in line with local research and ethics practice.

**Data collection:** Workshops will be held in locations convenient for participants and will be facilitated by experienced members of the research team with the assistance of local researchers.

The workshops will utilize a series of scripts as part of the data collection. Drawing on a collation of scripts developed by the team in previous studies of health systems resilience^[12]^, these will include a mix of rich pictures, graphs over time, variable elicitation, causal loop model development and refinement and potential priority-setting of system strengthening interventions. All exercises will be informed and adapted based on the document review and KIIs conducted for each setting, however a brief description follows:

1) We will use rich pictures to understand the context (taking into account: early phases of the displacement, compared to later phases of displacement and stabilization) in which the health system had to adapt and deliver services to refugees and identify how said adaptation occurred. Perceptions of the pressures and facilitators faced by diverse stakeholders will also be probed. In the case of community members, social cohesion will also be explored. Community members may be asked to elicit health seeking pathways and comment on the trust that they hold in local health care delivery stakeholders.

2) Graphs over time will provide insights into how the diverse GMB participants view key trends over time in specific outcomes (i.e. perceptions of health service delivery outputs, health outcomes, integration processes and their success/perceptions service satisfaction of host and refugee populations, perception of social cohesion).

3) Variable elicitation and refinement will focus on identifying the key influences on refugee service provision by health systems and the outcomes of interest to GMB participants. An initial set of variables for each site will be elaborated following the desk review and KIIs and then will be refined with the participants (feeding in insights gained from rich pictures and graphs over time). Variables could relate to health system resilience, effective and equitable responses, social cohesion, but will be prioritized by participants themselves.

4) Causal loop models will then be developed iteratively by the GMB participants, and refined in dialogic interaction with facilitators, to identify the mechanisms via which appropriate, equitable, effective and cost-effective and efficient services have been delivered, including consideration of health system resilience and inclusion and integration of refugees into health service delivery and systems more broadly.

5) Elicitation and priority-setting of mechanisms for response enhancement/strengthening: GMBs often elicit substantive learning surrounding potential mechanisms for strengthening of systems or existing interventions (be they responses overall or services). Similar information will also be probed for during interviews (e.g. lessons learned, what can be done better). Therefore informed by the findings of the KII, GMB participants will also be asked to reflect on the models they created and further elicit mechanisms which can target key points of failure or discontent within these. They will also be asked to elicit criteria for priority-setting surrounding these mechanisms (e.g. considering issues such as feasibility, sustainability, efficiency, resilience of health systems, cost-effectiveness), and further undertake said priority setting.

*Data analysis from GMB*

While the GMB approach adopted here is a mix of community-based system dynamics and more formalised system dynamic inquiry with expert participants^[13]^, the core set of system dynamics principles of analysis will be applied. GMB workshops generate a wealth of data, ranging from pictorial (rich pictures and graphs over time), audio (workshops are usually recorded), to a mix of thematic-visual (causal loop models). The latter will be analysed iteratively by the study team, following a system dynamics methodology, to identify the underlying dynamics driving refugee responses and their relative success in ensuring appropriate, equitable, effective and cost-effective care, while balancing sustainability of service delivery and current health system dynamics.

First, data cleaning will take place: variable names will be clarified by listening to audio recordings and variables will be redefined based on principles used in system dynamic analysis. Second, links between variables will be interrogated to identify whether variables are associated or causally linked, with links then refined and defined and causal loops identified. Loops will then be further abstracted and validated against the findings of the KIIs and data and desk review.

### Overall data analysis and write up

The data collected through different methods will then be processed and findings written up into a short report (one per case study).

The report will include elements as follows:

- Executive summary featuring consolidated learning and key findings from this case study
- Introduction to overarching study and methods
- Background country context: discussing the economic and political situation, including discourse on migration and refugees, health system and demographic background
- Refugee situation, and response timeline: Stage and history of displacement, how response is organised, demography and epidemiology of refugee population, what donors and NGOs are present, legal situation of refugees, including rights to work, other services
- Findings from each of the key elements of the conceptual framework, drawing from all methods:
  - Service provision
  - Governance
  - Financing
  - Monitoring, Evaluation and Learning
  - UHC outputs and outcomes
  - Impact on wider health system
  - Impact on social cohesion
  - Resilience, sustainability, efficiency and cost-effectiveness.
- Limitations and strengths of the work
- Implications and recommendations

A synthesis report will also be prepared bringing together, comparing and contrasting the findings from the six case studies. The key analytical focus here will be on answering the main research questions.

## Quality assurance

The study team is composed of staff from different research partners with complementary expertise, and different levels of seniority. Quality assurance will be pursued through linked strategies of writing clear protocol and topic guides, ensuring good communication between leads and researchers and between researchers in different countries or institutions, mentoring and capacity building of junior researchers throughout the process, through close support and oversight by more senior researchers and ensuring that all staff have appropriate skills and backgrounds, including for any additional local staff working on the project. Further, induction training on methods and tools will be provided prior to fieldwork, particularly addressing interviews virtually or at a distance. Robust organization of fieldwork with time and space for reflection on gender, equity and justice issues will be ensured. We will also conduct a rigorous process for transcription of the data and accurate translation of tools, transcripts and notes, where applicable. Regular debriefing meetings during fieldwork to review recordings, notes and transcripts will take place, where the team will suggest ways to improve quality including revision of topic guides and development of further probing questions, addressing any gaps and errors immediately.

Additionally, triangulation of findings across tools and data sources – looking for overlap and inconsistencies - will be ensured. Collaborative work on writing, including internal peer review process of written outputs (protocol, initial report, papers, s and/or policy briefs) will also support higher quality outputs.

To reduce bias during data collection and analysis, the process will be monitored. A random sample of interviews will be reviewed by a team leader to ensure the process is being carried out according to the protocol. The same will be done during the analysis stage, where different team members would cross check with one another to ensure data validity and reliability.

Respondent validation will be used whereby the data collectors double-check they understood the participants’ responses, reducing interpretative bias. This may be done by several techniques such as paraphrasing the respondent’s response to check the data collector understood the answer correctly, asking the participants if the question was clear, and re-assuring the participants that they can take their time in responding to questions. This is especially relevant for questions require recalling of events or numbers which happened in the past.

Finally, tools requiring translation will be back-translated to ensure the tool for data collection is understandable and accurate in the local language.

## Ethical considerations

*Personal information, anonymity and confidentiality*

The information collected from stakeholders and other research participants does not pertain to them personally, but refers to general views and opinions relating to their professional practice and their experience of the health system. Data will be anonymised and stored safely following GDPR guidelines. Only the main researchers and supervisors will have access to the stored data. No exact locations or geographical identifiers will be included to ensure full safety and privacy of participants. Data collection will be stopped if the participant becomes distressed. Participating or not participating will not have any effect on someone invited to engage in the research. Data collectors will be careful not to interrupt the routine (for example, in a health facility) of the participants when conducting the study. Interviews will be held during times suitable for the participant.

In some cases, participants may feel vulnerable working in difficult conditions, may be exhausted and under enormous stress. They may become distressed retelling their experiences working with few resources to provide care or under insecure conditions for them and their families. For the interviews and GMB workshops, data collection tools will be kept to a minimum and the data collectors will be trained on sensitivity towards respondents. The research team will be matched to the context as far as possible, to ensure that cultural sensitivities are taken into account and that the approach remains in line with local norms and values. Our approach is to support respondents and handle interviews sensitively and without judgement. The interviewers will stop the interviews and give participants time to recover and ask them to continue now or at a later date, or stop altogether. Sensitive phrasing of questions, showing empathy and observing for signs of distress will also be used to help participants recover from distressing events.

Some of the information gathered, for example in relation to views of state actors and international actors might be sensitive. In order to mitigate any potential adverse consequence for the respondents, the confidentiality of data and respondents will be essential to ensure no harm to respondents in retribution. Reporting will be done in a way that ensures anonymity of the respondents.

A consent sheet and a participant information sheet (PIS) will be signed by the participant, or, if not possible, orally approved by participant and registered by the interviewer. This consent sheet will be linked to the data via a numerical code. The PIS describing the aims of the study using simple language will be shared with participants who would be allowed enough time to ask questions prior to agreeing to participate in the interview or the workshops. The PIS will outline that participants can withdraw from the study at any time without any issue.

*Data management*

Hard copies of data containing personal information, such as consent forms and handwritten field notes, will be kept in locked storage in the office of a designated person in each institution. Raw data (audio files recordings) will be uploaded into channels within the QMU cloud system which only the research team has access to. All electronic devices used in the research process will be encrypted and password protected. Transcriptions and typed-up field-notes will be immediately anonymised and uploaded into channels within the QMU cloud system which only the research team has access to. They will also be kept on password-protected devices of the research team during analysis. The data storage platform will automatically back-up the data and data will be archived monthly. Only data that needs to be shared will be. For example, no partner beyond the in-country research team needs to see the consent forms or names and contacts details of participants, and therefore these kinds of data will not be shared with the other partners.

Anonymised electronic data will be stored by QMU for 3 years after the end of the project. Paper-based and electronic (raw) data will be stored by the research team for 3 months after the end of the study.

*Ethics approval*

The study protocol will be reviewed by QMU ethics committee – and suitable revisions made to secure approval - before the study implementation and subsequently, where relevant and needed, submitted for review by local ethics committees. Administrative authorizations will be sought with national, or regional/district bodies as required in each country. Relevant risk assessment forms, including travel risk assessments will be conducted for each country case study team.

## Research uptake and dissemination

The plans for research uptake and dissemination include academic publications, policy briefs, webinar/ dissemination workshops and a final report.

With UNHCR, we will collectively define key stakeholders for this programme, which is likely to include different sections and divisions within UNHCR, as well as wider actors (other UN organisations, bi- and multilateral funders, host governments, non-governmental organisations and civil society organisations). Strategies for engaging and communicating with different stakeholder groups will be developed as the case studies progress.

Research uptake and dissemination is expected to be at country level, for country case studies, as well as regionally and globally. Research uptake and dissemination will take the form of:

- Meetings with key stakeholders (in-country partners, community representatives, UNHCR local offices)
- Webinars to target a broader group of stakeholders at regional and global level
- Website content, through the IGHD and UNHCR webpages
- Policy and practice briefs for relevant actors/constituencies
- Documentation (and presentations, as appropriate) for relevant high-level UNHCR meetings (e.g. ExCom, High Commissioner’s Dialogue, etc.) and Global Refugee Forum briefings.
- Journal articles

Conferences as relevant, for example the International Conference on Refugee and Forced Migration Studies

- Public academic seminars to further academic debate on refugee-related research (such as the public seminar series at the Oxford University Refugee Studies Centre with which we have previously engaged)
- Social media for shorter messaging, including Twitter and LinkedIn and academic mailing lists (such as GRAMNet).

## Timeline

The following table shows a breakdown of the timeline of each individual case study. This will be adapted to each country case study, with a general timeframe of 9 months per case study

## Research team

From the QMU side, the research team is led by Professor Sophie Witter, who is the overall PI of the project. The core team liaises on a regular basis with UNHCR. Working closely with the management team is a group of researchers who will work on the case studies, and a group of experts who can support specific areas of methodology and analysis.

Country case studies will be led by a team of an international researcher(s) and one or multiple country researcher(s), depending on local arrangements and research partners. Research partners will be identified in all case study countries and their exact role will depend on their skills and expertise. In some case, they might work in close collaboration with the QMU country leads for data collection and analysis, and in other cases they will be supported remotely by the QMU team.

## Annex 1: Participant Information Sheets

### Participant Information Sheet - Interviews

My name is [Researcher name] and I am working on a research project with the Institute of Global Health at Queen Margaret University in Edinburgh, carried out on behalf of UNHCR. The project focuses on: **Health system resilience, enhancement and refugee responses.**

**You are being invited to take part in this study, but before you decide whether or not to take part, it is important for you to understand why the research is being done and what it will involve. Please take time to read the following information carefully. Talk to others about the study if you wish. Contact us if there is anything that is not clear or if you would like more information. Take time to decide whether or not you wish to take part.**

**What is the purpose of the study?**

This study considers how countries and health systems respond to the health needs of refugees, including looking at the wider impact of refugee displacement and settling on the country’s host population and systems in place.

**Why have I been asked to take part?**

You have been asked to take part as you have experience in responding to refugee’s health needs.

**Do I have to take part?**

No, it is up to you to decide whether or not to take part, participation is voluntary. If you do decide to take part, you will be given this information sheet to keep and be asked to sign a consent form. If you decide to take part, you are still free to withdraw at any time and without giving a reason and without any issue. Deciding not to take part or withdrawing from the study will not affect your legal rights or relationship with any institution.

**What will happen if I take part?**

If you *agree to participate,* you will be asked to read and sign a consent form. Where the interview is done remotely, there will be an option to take verbal consent and record using an app on the telephone, or an external recorder.

An interview will be carried out face-to-face but could be virtual if you prefer. It will be conducted at a time that is convenient for you and in the language that is most familiar for you and (in case of face to face) in a private and safe location. Interviews will last approximately one hour.

Within this, you will be asked a series of questions on the topic. You will be free to withdraw from the study at any stage and you will not have to give a reason and without any issue

**What are the possible benefits of taking part?**

Whilst the study might not benefit you directly, we hope that the findings from this study, can help inform the health response for refugees and to make it fairer and stronger for the population as a whole.

**What are the possible disadvantages and risks of taking part?**

It is not thought that there are any disadvantages, however, if there is any part of the data collection process, you are not comfortable with, please let us know straight away and we will ensure the correct steps are taken to ensure your safety and comfort is taken care of.

**What happens when the study is finished?**

Transcripts from the study will be anonymised and then analysed by the research team. Data will be transferred to the main research team at Queen Margaret University, in the UK. The findings will be published to inform future planning on the topic, but no personal identifiers will be included and your data will be entirely anonymous. All records of the study will be destroyed 3 years after the end of the study.

**Will my taking part in the study be kept confidential?**

All data will be anonymised, and your name will be replaced with a participant number, and it will not be possible for you to be identified in any reporting of the data gathered.

**GDPR Notification:**

All of your personal information will be treated in accordance with the terms of the UK Data Protection Act 2018 and the General Data Protection Regulation (GDPR). Processing your personal information is necessary for the performance of a public task carried out in the public interest or in the exercise of official authority vested in us as the Data Controller (GDPR Article 6 (1)(e) and for statistical and research purposes (Article 89 GDPR). Appropriate security measures including anonymisation will be put in place to protect your data at all times. Your personal data will be treated with confidentiality and will not be shared with any third party or transferred out with the EEA without your express permission or without ensuring appropriate safeguards are in place. Your data will only be retained for as long as is necessary. You have the right to withdraw your consent to us processing your personal data at any time. In order to do so, please contact Natasha Palmer (npalmer@qmu.ac.uk). Please note that your data may be used in the production of formal research outputs before you withdraw consent, therefore it is advisable to contact us as soon as possible if you wish to withdraw your consent. We will destroy your identifiable data upon request, where possible, however in some situations we will require to use the data collected up until your withdrawal of consent. If you have any questions relating to the processing of your data which are not resolved by contacting Natasha Palmer (npalmer@qmu.ac.uk) please contact the QMU Data Protection Officer [cdickson1@qmu.ac.uk](mailto:FOI@qmu.ac.uk)

**What will happen to the results of the study?**

The study will be written up as a final report, based on which other products such brief summaries of the research, conference presentations and journal publications will be created. No traceable information will be shared. We will additionally organise meetings and webinars to make participants and other key stakeholders aware of the findings.

**Who is organising the research and why?**

This study is led by Queen Margaret University in collaboration with [ADD collaborating external organisation(s)] and funded by the UNHCR.

**Who has reviewed the study?**

The study proposal has been reviewed by the QMU ethics committee and a favourable ethical opinion has been obtained [if applicable - add if requiring also ethics from an external ethics committee or abroad].

**If you have any further questions about the study please contact:**

Name of researcher: Dr Natasha Palmer

Address: IGHD

Queen Margaret University, Edinburgh

Queen Margaret University Drive

Musselburgh

East Lothian EH21 6UU

Email:	npalmer@qmu.ac.uk

**If you have any complaints about this study or would like to report an adverse event please contact:**

QMU University Research Ethics Committee

Address: QMU Research Ethics Committee

Queen Margaret University, Edinburgh

Queen Margaret University Drive

Musselburgh

East Lothian EH21 6UU

Email: [Researchethics@qmu.ac.uk](mailto:Researchethics@qmu.ac.uk)

**If you have read and understood this information sheet, any questions asked have been answered, and you would like to be a participant in this study, please now see the consent form.**

Thank you for taking the time reading this information sheet.

### Participant Information Sheet – GMB Workshops

My name is [Researcher name] and I am working on a research project with the Institute of Global Health at Queen Margaret University in Edinburgh, carried out on behalf of UNHCR. The project focuses on: **Health system resilience, enhancement and refugee responses.**

**You are being invited to take part in this study, but before you decide whether or not to take part, it is important for you to understand why the research is being done and what it will involve. Please take time to read the following information carefully. Talk to others about the study if you wish. Contact us if there is anything that is not clear or if you would like more information. Take time to decide whether or not you wish to take part.**

**What is the purpose of the study?**

This study considers how countries and health systems respond to the health needs of refugees, including looking at the wider impact of refugee displacement and settling on the country’s host population and systems in place.

**Why have I been asked to take part?**

You have been asked to take part as you have experience in responding to refugee’s health needs.

**Do I have to take part?**

No, it is up to you to decide whether or not to take part, participation is voluntary. If you do decide to take part, you will be given this information sheet to keep and be asked to sign a consent form. If you decide to take part, you are still free to withdraw at any time and without giving a reason and without any issue. Deciding not to take part or withdrawing from the study will not affect your legal rights or relationship with any institution.

**What will happen if I take part?**

The data collection will be face to face. Workshops will last 1 day and will be organised at a time that is most convenient for you. Workshops will be done in a safe location, and will be conducted in the language that is most familiar to you. Each workshop will have a series of activities that you will be asked to actively participate in.

You can withdraw from the study at any time, up to the point that workshop materials undergo anonymisation and analysis. After this, it will no longer be possible to identify and remove your inputs.

**What are the possible benefits of taking part?**

Whilst the study might not benefit you directly, we hope that the findings from this study, can help inform the health response, to make it fairer and stronger for the population as a whole.

**What are the possible disadvantages and risks of taking part?**

We encourage you not to reflect on personal but rather general community or professional experiences within workshops. However, we understand that this reflection may be distressing. Our research teams are trained in providing support and also signposting you to relevant support services.

**What happens when the study is finished?**

Transcripts from the study will be anonymised and then analysed by the research team. Data will be transferred to the main research team at Queen Margaret University, in the UK. The findings will be published to inform future planning on the topic, but no personal identifiers will be included and your data will be entirely anonymous All records of the study will be destroyed 3 years after the end of the study.

**Will my taking part in the study be kept confidential?**

All data will be anonymised, and your name will be replaced with a participant number, and it will not be possible for you to be identified in any reporting of the data gathered.

**GDPR Notification:**

All of your personal information will be treated in accordance with the terms of the UK Data Protection Act 2018 and the General Data Protection Regulation (GDPR). Processing your personal information is necessary for the performance of a public task carried out in the public interest or in the exercise of official authority vested in us as the Data Controller (GDPR Article 6 (1)(e) and for statistical and research purposes (Article 89 GDPR). Appropriate security measures including anonymisation will be put in place to protect your data at all times. Your personal data will be treated with confidentiality and will not be shared with any third party or transferred out with the EEA without your express permission or without ensuring appropriate safeguards are in place. Your data will only be retained for as long as is necessary. You have the right to withdraw your consent to us processing your personal data at any time. In order to do so, please contact Natasha Palmer (npalmer@qmu.ac.uk). Please note that your data may be used in the production of formal research outputs before you withdraw consent, therefore it is advisable to contact us as soon as possible if you wish to withdraw your consent. We will destroy your identifiable data upon request, where possible, however in some situations we will require to use the data collected up until your withdrawal of consent. If you have any questions relating to the processing of your data which are not resolved by contacting Natasha Palmer (npalmer@qmu.ac.uk) please contact the QMU Data Protection Officer [cdickson1@qmu.ac.uk](mailto:FOI@qmu.ac.uk)

**What will happen to the results of the study?**

The study will be written up as a final report, based on which other products such a brief summaries of the research, conference presentations and journal publications will be created. No traceable information will be shared. We will additionally organise meetings and webinars to make participants and other key stakeholders aware of the findings.

**Who is organising the research and why?**

This study is led by Queen Margaret University in collaboration with [ADD collaborating external organisation(s)] and funded by the UNHCR.

**Who has reviewed the study?**

The study proposal has been reviewed by the QMU ethics committee and a favourable ethical opinion has been obtained [if applicable - add if requiring also ethics from an external ethics committee or abroad].

**If you have any further questions about the study please contact:**

Name of researcher: Dr Natasha Palmer

Address: IGHD

Queen Margaret University, Edinburgh

Queen Margaret University Drive

Musselburgh

East Lothian EH21 6UU

Email:	npalmer@qmu.ac.uk

**If you have any complaints about this study or would like to report an adverse event please contact:**

QMU University Research Ethics Committee

Address: QMU Research Ethics Committee

Queen Margaret University, Edinburgh

Queen Margaret University Drive

Musselburgh

East Lothian EH21 6UU

Email: [Researchethics@qmu.ac.uk](mailto:Researchethics@qmu.ac.uk)

**Procedure for safeguarding/signposting:**

*In case you experience distress during this workshop or wish to speak to someone about a safeguarding concern please contact: [INFO per country]*

**If you have read and understood this information sheet, any questions asked have been answered, and you would like to be a participant in this study, please now see the consent form.**

Thank you for taking the time reading this information sheet.

## Annex 2: Consent form

### CONSENT FORM FOR PARTICIPANT

Title of Project: Health system resilience, enhancement and refugee responses project

Name of Researcher: [Researcher name]

| **Circle yes or no for each statement below** | | | | | |
| --- | --- | --- | --- | --- | --- |
| 1. I confirm that I have read and understand the participant information sheet dated 14^th^ of June 2023, for the above study. I have had the opportunity to consider the information, ask questions and have had these answered satisfactorily. | | | | | **YES / NO** |
| 2. I understand that my participation is voluntary and that I am free to withdraw at any time without giving any reason. Withdrawing will not affect any of my rights. | | | | | **YES / NO** |
| 3. I agree for data collected to be used in future ethically approved research. | | | | | **YES / NO** |
| 4. I agree for my data to be transferred and processed in the UK. | | | | | **YES / NO** |
| 5. I agree to be audio recorded | | | | | **YES / NO** |
| 6. I agree to take part in the above study. | | | | | **YES / NO** |
|  |  |  |  |  | |
| Name of Participant |  | Date |  | Signature | |
|  |  |  |  |  | |
| Name of Person taking consent |  | Date |  | Signature | |

## Annex 3: General topic/interview guide for key informant interviews

**Health system resilience, enhancement and refugee responses project**

***General topic guide - To be adapted for each country case study***

*Note details of participants before interview:*

| 1. Interviewee ID |  |
| --- | --- |
| 2. Date of Interview |  |
| 3. Gender | Male Female |
| 4. Title of interviewee |  |
| 5. Institution / Organization / Department |  |
| 6. Location of interview |  |

- *Confirm that information sheet has been read and understood,*
- *Ask for any further questions*
- *Check that consent form has been signed*
- *Check/ask for consent for recording the interview.*

*Remember to adapt and tailor questions according to (i) specific needs/gaps in information identified during the document review and data analysis stages (some of the questions in the guide might be already covered by document review), (ii) background of the key informant, their experience and potential knowledge. Also note that ordering of questions can be rearranged.*

*Remember to ask for further documentation, data and suggestions for other key informants.*

1. Could you please tell me what is your **current professional role**? Which part of the refugee response you have been involved in? For how long? Have your role and position changed overtime? (if so, explain)
2. How is **healthcare for refugees broadly organised** in [site or country]?

Probe by asking about the following – *note that we might know much of this from the doc review so check specifically for gaps in information*:

- Modalities of healthcare provision (approaches, packages of services, pathways and referrals, adequacy of health staff and integration of refugee health staff, ...)
- Governance and coordination: who are the main actors involved in the provision/organisation of healthcare for refugees?, what are their roles? How do coordinated they are, and how/through which mechanisms? How effective do you think the coordination is?
- Health financing arrangements [*broadly we are interested in sources of financing, pooling and purchasing arrangements, benefit packages – but we might have most of this from doc review. Probe for gaps in understanding, specificities at local level, changes over time/see next question*]
- MEL: how is the routine health information system working? - type of data collected, for whom (refugee-specific, general pop, etc.), at which level, by whom, analysed and used?, how?); are there any surveys being done recently/planned? Which population(s) are captured in the surveys?

3. What have been the main **changes over time** [*against elements in question (2), probe using those*], and why/what triggered the changes?

If no changes: what reasons or elements in the situation and response, do you think led to the decision to organised healthcare for refugees in such way as described before?

[*here we are aiming to understand how context influences healthcare provision for refugees – do reframe/reformulate if helpful for the respondent*]

4. How do you think the way healthcare for refugees is organised impacts on **health outputs and outcomes** such as coverage of services (i.e. the services available for the refugees), quality of services, accessibility/equity (which means whether everyone can access services based on their needs), health outcomes (by which we mean the results in terms of health and wellbeing levels)

- Could you provide examples or explanations of this? Could you explain what you think is the link or pathways between how healthcare is organised and those outputs/outcomes?

5. What do you think are the main **challenges** in healthcare provision for refugees? And on the other hand, what is **working well**? Why so (in both cases)?

Probe:

- for each of the elements in question (2)
- ask to make examples

Further questioning:

- what do you think are the main issues or areas where approaches should be changed, and why?
- What are the lessons learned from changes or adjustments made in the past?
- What are the trade-offs in relation to changes and adjustments to address challenges? By which we mean, what is the balance between those adjustments? What needs to be given up to address one given issue?

6. We want to understand better the level of **integration** of refugees in the local health system, by which we mean whether refugees have access to the local/national health system and services (or to some elements of that) under the same conditions as nationals.

- [First of all, could you describe broadly what the local health system looks like? What are the main challenges and constraints, and the opportunities?]
- In relation to healthcare for refugees specifically, what is integrated and what is not - and what is the level of integration [*probe using elements in question (2) above, but respondents could also identify other elements or frame differently*]
- In your view, what is working well and what is not working in the integration of refugees in the local health system?
- Looking at the future, what could be (better or more) integrated? And what should not be integrated? Why?

7. How to do think the current organisation of healthcare for refugee works in relation to:

- **Sustainability** – by which we mean can it be maintained over time? If not, what would be the main challenges in the future? Please, make specific examples
- **Efficiency and cost effectiveness**, by which we mean whether it uses the least possible (financial) resources to achieve the best possible results. Again, could you make specific examples? Could you explain in which sense arrangements are achieving (or not) efficiency and cost-effectiveness? In which areas?

8. What do you think is the impact of how healthcare for refugee is organised on **social cohesion** and the relations between refugees and local (non-displaced) communities?

- Do you have any practical, concrete examples from your own experience to support your views?

[at the beginning or end]: Check for any contextual information or gap in understanding that we have based on document review and data analysis. It might be covered in the above questions/answers, but make sure all information gaps are addressed.

***END OF INTERVIEW***

- *Thank participant for their time.*
- *Remember to ask for further documentation, data and suggestions for other key informants*
- *Share information and contacts for longer-term engagement and dissemination of results.*

## Annex 4: Outline of Group model building workshop

**Health system resilience, enhancement and refugee responses project**

***General workshop guide - To be adapted for each country case study***

**Duration: 1 day**

**Facilitation: 1 modeller, 2 group facilitators, 1 observer**

| **Duration** | **Exercise description** |
| --- | --- |
| 30 minutes | **Introduction and welcome**  Participants introduce themselves, the research group introduces themselves and the project.  Ethical principles (right to confidentiality, anonymity and privacy) are reiterated.  Rules for the day are noted by the facilitator: respectful engagement, verbalise views as commonly expressed in community (no need to self-identify), no need for consensus/diversity of views encouraged. |
| 20 minutes | **Hopes and fears**  Participants are provided with post-it notes and are encouraged to note 1 hope and 1 fear they have in relation to the workshop and its stated aims. Facilitator discusses how the research team will remain accountable towards the participants. |
| 30 minutes | **Rich pictures**  Facilitator provides a flipchart with three delineated areas representing the acute emergency phase (when displaced), immediately post-displacement, and long term phase.  **Option for workshop 1 below, unlikely to be used in Workshop 2.**  For community members: Participants are urged to draw: how they sought care/how they received health care, how they felt received by communities and systems, who they felt were safe/trusted contacts to approach.  For health care providers: Participants are urged to draw: main challenge or problem for each phase of the response regarding care delivery, how they felt they were meeting needs of host and refugee communities, how confident/satisfied they felt regarding meeting needs.  Following elaboration of pictures, groups will be asked to present to one another and discuss differences in perspectives and perceptions. |
| 30 minutes | **Graphs over time**  Facilitator provides flipchart with a timeline that corresponds to the refugee response (1-2 years before, until current). The KIIs will inform the choice of specific graphs that participants are asked to draw, but examples are provided below.  **Option for workshop 1:**   - Availability of services for different communities and ability to access care; - Quality of care; - Health service capacity to deliver services (participants can choose to operationalise this further – e.g. capacity or availability of staff)   **Option for workshop 2:**   - Funding availability for health response - Coordination challenges and pressures regarding response - Availability and accessibility of services, health outcomes |
| 30 minutes | **Variable elicitation**  For all workshops: participants will be asked to look back at their rich pictures/graphs over time and identify key variables which impact on the provision of health services to refugees. They will also be asked to reflect on what constitutes success and identify variables associated with this - e.g. equity of provision between communities, social cohesion, sustainable service delivery/response. Based on KIIs, additional variables will be suggested for consideration. All variables will be written on post-it notes, their meanings clarified and then prepared for the next exercise. |
| 60 minutes | **Lunch break** |
| 90 minutes | **Causal loop diagram development**  During the lunch break, facilitators will prepare additional flipcharts and will ask participants to divide into groups (the characteristics of the division can be decided on the day). Participants will be asked to choose 10-15 variables from the ones previously developed to base their models on. The facilitator will then illustrate to participants how causal linkages are drawn and will prompt the groups to start working on their models. The facilitator will be moving between groups, prompting participants to clarify their thinking, and prompting for further information where needed. Groups will be asked to present their thoughts to one another. |
| 40 minutes | **Elicitation and priority-setting of mechanisms for response strengthening**  Based on the models drawn, participants will be asked to indicate where single points, or multiple pathways/dynamics of failure exist. They will be prompted to identify what actions could address these and priority-setting of said mechanisms will further be discussed. The KIIs will help identify and clarify priority setting criteria (e.g. sustainability, equity among others) that will help guide the process. |
| 20 minutes | **Close**  Researchers explain next steps for model cleaning and development and discuss the role of participants in any post-workshop validation activities. |

Annex 5: Data sources

Searching datasets from various international and national sources is a crucial step in conducting research on access to healthcare for refugees. The availability of diverse and comprehensive datasets can provide valuable insights into the topic.

International Sources: International organizations such as the United Nations, World Health Organization (WHO), International Organization for Migration (IOM), and UNHCR (United Nations High Commissioner for Refugees) are important sources for accessing relevant datasets on refugees and healthcare. These organizations often collect and maintain comprehensive datasets that cover various aspects of refugee populations, including healthcare access. Their websites, data portals, and publications will be explored to identify datasets on refugee health, healthcare services, health infrastructure, and related indicators. These sources may offer global or regional datasets, enabling researchers to gain insights into the broader context of refugee healthcare access.

National Sources: National government agencies, ministries of health, statistical offices, and research institutions of countries hosting refugees are valuable sources for accessing national-level datasets. These sources often provide data on healthcare services, utilization patterns, health outcomes, and policies specific to the host countries. National statistical databases and open data portals are useful starting points for searching for relevant datasets. Additionally, academic institutions and research centres working on refugee health may have their own datasets or repositories that researchers can explore. Collaborating with local researchers or engaging in partnerships with national stakeholders can facilitate access to valuable datasets and foster data sharing initiatives.

International Surveys: International surveys conducted by organizations like the WHO, IOM, or other research entities often include modules or sections on health and healthcare access for refugees. Examples include the Demographic and Health Surveys (DHS), Multiple Indicator Cluster Surveys (MICS), and Global Burden of Disease (GBD) studies. These surveys provide standardized data collection instruments and methodologies, allowing for cross-country comparisons and analysis. Exploring survey databases, survey-specific websites, or contacting the survey administrators can help researchers identify relevant datasets and access survey data for further analysis.

Data Sharing Platforms: Various data sharing platforms and repositories can serve as valuable resources for accessing datasets related to refugee healthcare access. Platforms like Data.gov, [Data.UNHCR.org](http://data.unhcr.org/), and the World Bank's Open Data initiative offer a wide range of datasets on assorted topics, including healthcare, refugees, and migration. Additionally, academic repositories and data archives, such as the Inter-university Consortium for Political and Social Research (ICPSR) or the UK Data Service, may contain relevant datasets contributed by researchers worldwide. These platforms often provide search functionalities and detailed metadata descriptions to facilitate dataset discovery and access.

Research Networks and Collaborative Projects: Engaging with research networks and collaborative projects focused on refugee health and healthcare access can provide access to specialized datasets and foster collaborations. Organizations like the Migration Data Portal, Mixed Migration Centre, or academic networks dedicated to refugee and migration studies often curate datasets and facilitate data sharing among researchers.

It is essential to thoroughly evaluate the quality, reliability, and relevance of the identified datasets to ensure their suitability for our objectives.

1. United Nations. (2018). Global Compact on Refugees.
2. IASC and UN Working Group on Transitions. (2016). Background paper on Humanitarian-Development-Peace Nexus.
3. United Nations. (2018). Global Compact on Refugees.
4. UNHCR (nd) Access to Healthcare - <https://www.unhcr.org/uk/what-we-do/safeguard-human-rights/public-health/access-healthcare>
5. [World Bank Consortium : The Big Questions in Forced Displacement and Health](https://documents.worldbank.org/en/publication/documents-reports/documentdetail/099001106212246276/p1669090e0c4010b0adfa050973837275a)
6. QMU/ UNHCR Literature review on Health system resilience, enhancement and refugee responses. Unpublished report.
7. Gilson, L., Hanson, K., Sheikh, K., Agyepong, I.A., Ssengooba, F., Bennett, S., 2011. Building the Field of Health Policy and Systems Research: Social Science Matters. PLoS Med 8, e1001079. <https://doi.org/10.1371/journal.pmed.1001079>

   .
8. [Saturation in qualitative research: exploring its conceptualization and operationalization - PMC (nih.gov)](https://www.ncbi.nlm.nih.gov/pmc/articles/PMC5993836/#:~:text=Saturation%20means%20that%20no%20additional,that%20a%20category%20is%20saturated.)
9. Ritchie, J., Spencer, L., & O’Connor, W. (2003). Carrying out qualitative analysis. *Qualitative research practice: A guide for social science students and researchers*, *2003*, 219-62.
10. Thomas, J., Harden, A. Methods for the thematic synthesis of qualitative research in systematic reviews. *BMC Med Res Methodol* **8**, 45 (2008). https://doi.org/10.1186/1471-2288-8-45
11. Vennix, J. A. M. (1996). Group Model Building: Facilitating Team Learning Using System Dynamics. *Journal of the Operational Research Society*, *49*, 312
12. https://www.rebuildconsortium.com/resources/scripts-to-support-group-model-building-a-guide-for-participatory-systems-analysis/
13. Hovmand: community system dynamics and can also quote this:

    https://academic.oup.com/heapol/article/37/10/1328/6654776
